# Aging‐associated decline in vascular smooth muscle cell mechanosensation is mediated by Piezo1 channel

**DOI:** 10.1111/acel.14036

**Published:** 2023-11-09

**Authors:** Ngoc Luu, Apratim Bajpai, Rui Li, Seojin Park, Mahad Noor, Xiao Ma, Weiqiang Chen

**Affiliations:** ^1^ Department of Biomedical Engineering New York University Brooklyn New York USA; ^2^ Department of Mechanical and Aerospace Engineering New York University Brooklyn New York USA; ^3^ Laura and Isaac Perlmutter Cancer Center NYU Langone Health New York USA

**Keywords:** aging, calcium, cellular senescence, cytoskeleton, mechanobiology, smooth muscle cells, vascular smooth muscle

## Abstract

Aging of the vasculature is associated with detrimental changes in vascular smooth muscle cell (VSMC) mechanosensitivity to extrinsic forces in their surrounding microenvironment. However, how chronological aging alters VSMCs' ability to sense and adapt to mechanical perturbations remains unexplored. Here, we show defective VSMC mechanosensation in aging measured with ultrasound tweezers‐based micromechanical system, force instantaneous frequency spectrum, and transcriptome analyses. The study reveals that aged VSMCs adapt to a relatively inert mechanobiological state with altered actin cytoskeletal integrity, resulting in an impairment in their mechanosensitivity and dynamic mechanoresponse to mechanical perturbations. The aging‐associated decline in mechanosensation behaviors is mediated by hyperactivity of Piezo1‐dependent calcium signaling. Inhibition of Piezo1 alleviates vascular aging and partially restores the loss in dynamic contractile properties in aged cells. Altogether, our study reveals the signaling pathway underlying aging‐associated aberrant mechanosensation in VSMC and identifies Piezo1 as a potential therapeutic mechanobiological target to alleviate vascular aging.

## Graphical abstract

Aging is associated with a detrimental decline in vascular smooth muscle cell mechanosensation. Aged vascular cells exhibited a more inert mechanobiological phenotype due to cytoskeletal stiffening. The overexpression of Piezo1 ion channels and its calcium signaling is a key regulator of the aberrant mechanosensation in aging.

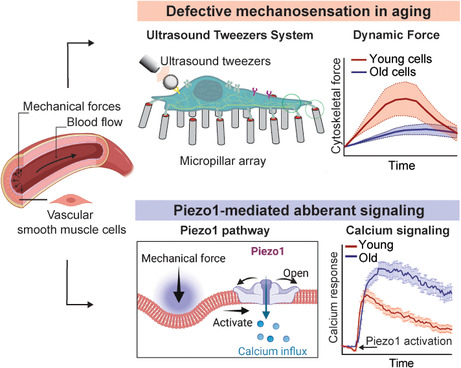

**Abbreviations**
AngII: angiotensin II
Ca^2+^
: calcium ion
CSK: cytoskeleton
ECM: extracellular matrix
EMD: empirical mode decomposition
F‐actin: actin filaments
GO: gene oncology
HHT: Hilbert‐Huang transform
IMFs: intrinsic mode functions
KEGG: Kyoto encyclopedia of genes and genomes
NF‐κB: nuclear factor‐κB
PDMS: polydimethylsiloxane
qRT‐qPCR: quantitative reverse transcription PCR
RGD: Arg‐Gly‐Asp
scRNA‐seq: single‐cell RNA sequencing
SM‐MHC: smooth muscle myosin heavy chain
UMAP: uniform manifold approximation and projection
VSMC: vascular smooth muscle cell
α‐SMA: smooth muscle‐alpha actin
γ‐SMA: smooth muscle‐gamma actin

## 1 INTRODUCTION

Aging is a major risk factor for cardiovascular diseases, such as atherosclerosis, aneurysms, atrial fibrillation, hypertension, and cardiac hypertrophy (Lind et al., 2018). At both tissue and cellular levels, aging develops with detrimental changes in the structure and function of the vasculature, attributing to a notable decline in mechanical properties of vascular cells (Yamashiro & Yanagisawa, 2020). The aging of arterial wall impairs the natural ability to adapt to the rapidly changing mechanical stress (e.g., shear stress and cyclic stretch) exerted by blood flow. As the major motivators of aorta, vascular smooth muscle cells (VSMCs) are crucial in regulating the contractility of vessel walls and responding mechanical stress (Brozovich et al., 2016). Thus, a healthy VSMC mechanosensation behavior (the capability of sensing and transducing mechanical forces into intracellular signals) enables the vessels to accommodate the rapidly changing mechanical perturbations in arterial microenvironment. Stiffening of VSMCs and dysfunctions in VSMC mechanosensation are often hallmarks of cardiovascular disease development (Bajpai et al., 2021). Previous efforts of examining static mechanical properties of VSMC, such as stiffness and contractile force (Seawright et al., 2018; von Kleeck et al., 2021), failed to reveal the aging‐associated disease pathogenesis and progression mechanisms. Thus, understanding of the regulatory mechanism of abnormal VSMC mechanosensation during aging and its role in aging‐associated cardiovascular diseases is critically needed yet currently limited.

Cellular mechanosensation involves both mechanosensitive ion‐gated channels or surface ligands to sense the extracellular mechanical cues, and actomyosin cytoskeleton (CSK) as a key mediator to generate intracellular mechanical forces and transmit such signals to the downstream mechano‐responsive components (Garoffolo & Pesce, 2019). VSMC mechanosensation depends on the swift opening of Piezo1, a tension‐gated ion channel that allows the influx of calcium (Ca^2+^) ions upon activation to regulate vascular development, blood pressure, and hypertension‐dependent arterial remodeling (Yin et al., 2022). Recent studies identified Piezo1 as the culprit for VSMC maladaptive mechanosensation in aortic aneurysm, a lethal vascular disease that predominantly affects the aged population (Qian et al., 2022). However, whether the same underlying mechanism occurs in chronological or “normal” aging remains unclear. In contrast to pathological aging caused by diseases, chronological aging is characterized by a more gradual and progressive decline in the cells' functional properties during the maturational processes (Holloszy, 2000). Thus, the downstream effectors of Piezo1 activation that are associated with the advance of aging in VSMCs still requires further exploration. Due to the dynamic nature of the CSK machinery, it plays a critical role in the regulation of mechanosensation of cells to mechanical stimulus (Weng et al., 2016). Dysfunctions in VSMC actomyosin architecture, CSK contractile function, and mechanosensation have been implicated in aging‐associated vascular diseases such as aortic aneurysm (Milewicz et al., 2017). Nevertheless, unraveling the changes and interplays between the Piezo1 mechanosensitive channels and CSK integrity, resulting in pathological mechanosensation in vascular aging, demands further investigations.

Here, we applied a novel ultrasound‐tweezers‐based micromechanical system to study the changes in VSMCs' mechanosensation associated with aging. The integrated ultrasound‐tweezers system, together the elastic micropillar‐based traction force measurement and instantaneous frequency spectrum analysis, enabled us to apply a transient mechanical force to single cell along with temporal analysis of VSMC mechanosensation behaviors. The mechanobiological study revealed that aged VSMCs are associated with a “rigidified” mechanical state, in which they fail to rapidly sense and adapt to the mechanical perturbation from the ultrasound tweezers. The decline in mechanosensitive behaviors in aged cells was mediated by hyperactivity of Piezo1‐dependent calcium signaling. Determining the molecular drivers of altered mechanosensation behaviors of aged VSMC could provide promising targetable mechanobiological signals that could repress aging‐associated cardiovascular dysfunctions and disease progression.

## 2 RESULTS

### 2.1 Defective VSMC mechanosensation in aging measured with ultrasound tweezers‐based micromechanical system

**FIGURE 1:**
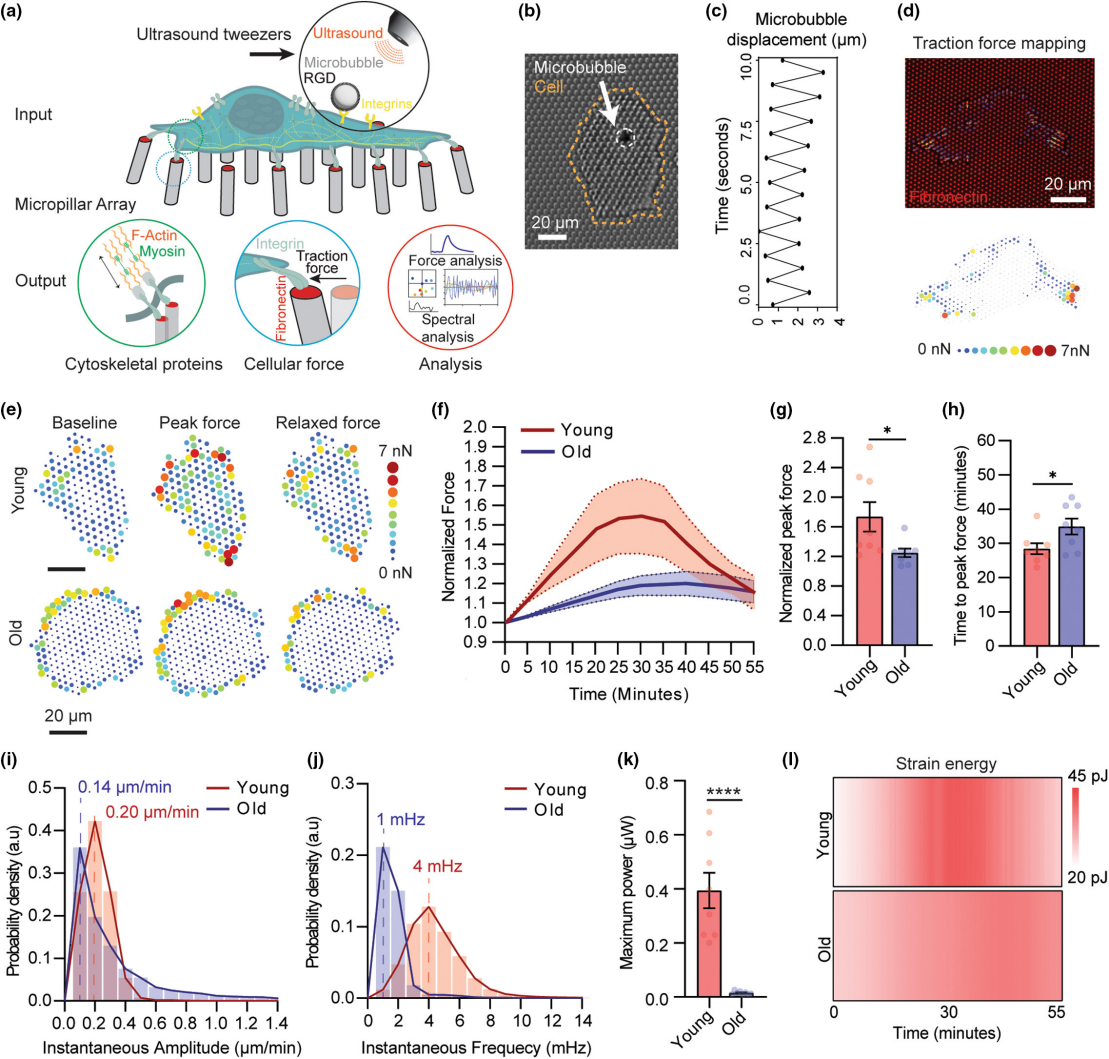
Defective VSMC mechanosensation in aging measured with ultrasound tweezers‐based micromechanical system. (a) A schematic of the integrated single‐cell micromechanical measurement system outlining the mechanical input, measurement, and output signals. The ultrasound‐tweezers system, together the elastic micropillar‐based traction force measurement and force instantaneous frequency spectrum analysis, allowing application of transient mechanical forces to single cell along with temporal analysis of VSMC mechanosensation behaviors. (b) A representative bright‐field microscopy image showing ultrasound microbubble attachment on VSMC on a PDMS micropillar substrate. VSMC is delineated by dotted orange line, microbubble is shown by white arrow. (c) Quantified microbubble displacement generated by the ultrasound tweezers under 1 Hz ultrasound stimulation over a period of 10 s. (d) Mapping of traction force of VSMC cultured on a fibronectin‐functionalized PDMS micropillar substrate. The top panel shows a representative fluorescent image of micropillar displacements (red channel) and quantified traction force vector map of VSMC (arrows). The bottom panel shows a traction force heatmap. (e) Representative heatmaps of dynamic traction force evolution of young and old VSMCs in response to a transient mechanical stimulation. Figures show forces prestimulation, force during peak contraction, and force 10 min post peak contraction. (f) Normalized global traction force in young and old VSMCs evolve after the transient mechanical stimulation. The thick line represents the mean of traction forces over time and the filled area represents the error bar presented as standard error of the mean (SEM). (g) Normalized peak force and (h) average time that each cell group took to reach peak force upon ultrasound tweezers stimulation. (i) Instantaneous amplitude and (j) instantaneous frequency distributions of force dynamic responses of young and old VSMCs from 0 to 30 min upon ultrasound tweezers stimulation. The thick lines represent mean amplitude and mean frequency response of young and old cells. (k) Maximum time‐averaged power supplied to single micropillar by young and old VSMCs upon ultrasound tweezer stimulation. (l) Representative heatmap of average strain energy in young and old cells in response to ultrasound tweezers stimulation. Data presented in f‐l are *n* = 8 cells per group. In g, h, and k, data are presented as mean values ± SEM, *p* values were calculated using Student's *t* test. *p < 0.05, ****p < 0.00005.

To evaluate the aging‐induced defective mechanosensation in VSMCs, we engineered a sophisticated single‐cell micromechanical system (Figure 1a, Figure S1) consisting of an ultrasound tweezers mechanical stress stimulator and an elastic micropillar array force sensor to apply a transient mechanical force to single VSMC and conduct in situ temporal measurements of their mechanoresponses to the mechanical perturbation (Qian & Chen, 2019). First, polydimethylsiloxane (PDMS)‐based micropillar arrays were manufactured using a standard soft lithography technique (Yang et al., 2011) and used as substrates and cellular force sensors (Figure S2; details see Appendix S1). The PDMS micropillar array substrates were microcontact‐printed with fibronectinand fluorescence‐tagged fibrinogen adhesive proteins on the top of the micropillars (Figure S2B). After cells were cultured on the micropillars, deflections of the micropillars underneath cells were continuously recorded under an inverted fluorescence microscope and used to quantify the forces exerted by the cells (Figure 1b, Figure S3) (Lendenmann et al., 2019). Ultrasound tweezers was used to apply a 10 s, 1 Hz, ~100 pN of transient force to single VSMC through an Arg‐Gly‐Asp (RGD)‐integrin bonded lipid‐encapsulated microbubble on the cell membrane (Figure 1c, Figure S4; details see Appendix S1) under ultrasound excitation, simulating an external mechanical stress (Q. Wang et al., 2019). In the ultrasound tweezers system (Figure S1), ultrasound pulses generated acoustic radiation force on the microbubble causing its displacement (Video S1), and therefore applied a controllable mechanical stress on the cell.

We applied the integrated ultrasound tweezers system to measure mechanosensation responses of primary VSMCs isolated from young (12 weeks) or old (58–78 weeks) mice to a transient mechanical stress on the cell membrane. Following the 10‐s exposure to stimulus, for both young and old VSMCs, traction force exhibited a biphasic feature, in which the single‐cell level traction force increased within the reinforcement period and gradually recovered in the relaxation period to reach mechano‐homeostasis as depicted by the force map of individual cells and quantification of normalized force over the 55‐min observation period (Figure 1e‐h). No significant change in force was observed in unstimulated cells (Figure S5A). The temporal analysis of force responses revealed that old VSMCs showed compromised ability to generate mechanoallostasic force compared to the young cells (Figure 1f). On average, young VSMCs' traction force increased 57% compared to the baseline force in the reinforcement period, while the old cells increased only 20% in response to the mechanical stress stimulation (Figure 1g). Meanwhile, old cells not only contracted less, but they also showed a slower and more prolonged contractile phase. The young cells reached their peak contraction within 27.0 ± 2.3 min, while the old cells reached the peak contraction in 34.8 ± 6.6 min after the stimulation (Figure 1h) and failed to reach the basal force level during the 55‐min observation period.

To further delve into the convoluted mechanical signals and analyze the aging‐associated shift in VSMC mechanosensation, we performed an instantaneous frequency spectrum analysis based on the single‐cell spatiotemporal force measurements, which has been shown to be able to effectively capture sensitive changes in subcellular dynamics across time and space. The force frequency spectrum analysis was based on the Hilbert‐Huang transform (HHT), an empirical method that has been successfully applied to explain nonlinear and nonstationary signal (Huang et al., 2003). We first performed empirical mode decomposition (EMD) to decompose the time series capturing velocity of individual micropillars into a finite and small number of modes called intrinsic mode functions (IMFs) to reduce noise in the original signal (Figure S6; details see Appendix S1). Application of the HHT to a specified IMF derive the instantaneous amplitude *A*(*t*) and frequency *F*(*t*), so called the instantaneous spectrum that provides frequency magnitude and distribution of the input force dynamics data. Physiologically, the instantaneous amplitude *A(t)* characterizes the magnitude of the force exerted by the cells to move the micropillars, while the instantaneous frequency *F*(*t*) characterizes the rate of which the actomyosin complexes are activated to generate traction force when the cells are subjected to a mechanical stimulation. A higher instantaneous frequency indicates more robust (active) actomyosin CSK dynamics and a higher mechanosensitivity. Thus, the instantaneous spectrum analysis provided a novel approach based on adaptive decomposition of local instantaneous force dynamics of single cells to distinguish spectral difference, and therefore to determine mechanosensation levels among cells. To validate the association of the instantaneous frequency and the CSK activity, we treated young VSMCs with 50 μM of Blebbistatin (Millipore Sigma) for 30 min to inhibit myosin‐mediated contractility before ultrasound tweezers stimulation. Blebbistatin‐treated VSMCs with inhibited CSK contractility showed a decrease in instantaneous amplitude and frequency compared to the control group (Figure S5B–D), suggesting that the force frequency spectrum analysis can pinpoint the changes in CSK‐associated force dynamics.

Upon instantaneous spectrum analysis of young and old VSMCs' force dynamic responses to the mechanical stimulation, we found that the old cells displayed an abate mechanosensitivity comparing to the young cells, indicated by decreases both in instantaneous amplitude *A*(*t*) (0.06 μm/min) and response frequency *F*(*t*) (3 mHz) (Figure 1i, j). The activation power derived from the instantaneous amplitude and frequency revealed the detrimental effect of aging on VSMC spontaneous force‐generating capacity, where the maximum power produced by old cells to move single micropillar were significantly lower than the young cells (Figure 1k). Such changes of VSMC mechanosensation were further specified by generating a temporal heatmap of VSMCs' strain energy response to reveal the decrease in strain energy consumed by old VSMCs during mechanical maladaptation (Figure 1l) (Qianbin Wang et al., 2019). Altogether, we characterized mechanosensation of VSMCs based on their dynamic force responses and the instantaneous frequency spectrum analyses and revealed a maladaptive mechanosensation in old VSMCs.

### 2.2 Loss of contractile phenotype and cytoskeletal integrity in VSMCs with aging

**FIGURE 2:**
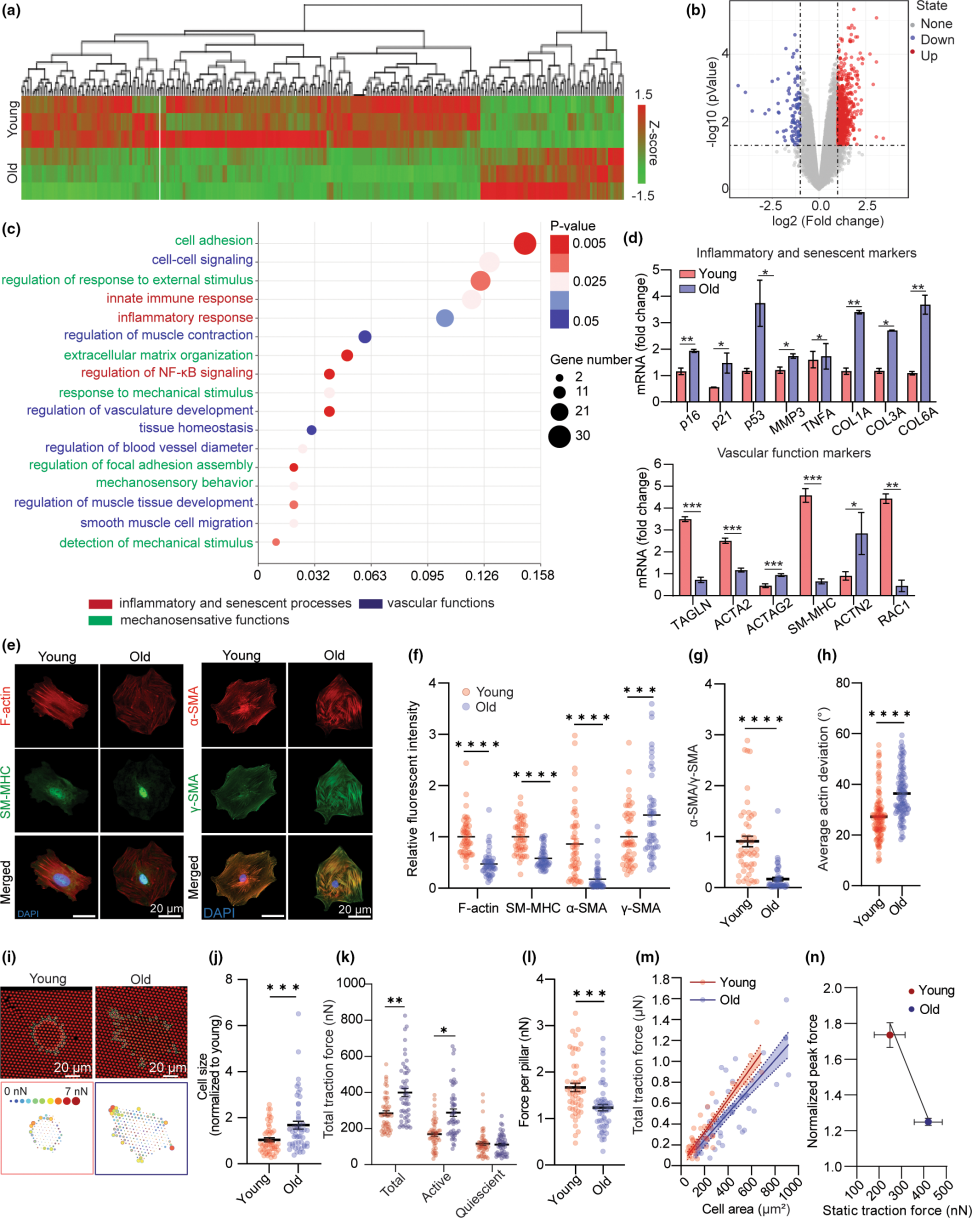
Loss of contractile phenotype and cytoskeletal integrity in VSMCs with aging. (a) Global gene profiles of young and old VSMCs by DNA microarray analysis (*n* = 3). (b) Volcano plot showing gene expression patterns of all VSMC samples. (c) Enriched pathways in old VSMC analyzed by GO Enrichment Pathway Analysis. Pathways related to VSMC functional behaviors are highlighted in colors. (d) qRT‐PCR profiled gene expressions related to VSMC functions and senescence in young and aged VSMCs. (e) Representative immunofluorescence staining images of F‐Actin, SM‐MHC, α‐SMA, and γ‐SMA in young and old VSMCs as indicated. DAPI is colored blue in merged images. (f) Quantification of critical cytoskeletal proteins: F‐Actin, SM‐MHC, α‐SMA, and ϒ‐SMA in young and old VSMCs. Fluorescent intensity of old cell group was normalized to the average intensity of young VSMC group (*n* = 50). (g) Ratio of α‐SMA/ϒ‐SMA showing relative expression of contractile Actin isoform over stiffness‐maintaining Actin isoform in young and old VSMCs (*n* = 50). (h) Quantification of the average Actin deviation of young and old VSMCs as indicated. *n* > 1000 square regions in both groups. (i) Representative fluorescent images showing micropillar displacements (top panel). (j) Quantified cell area, (k) total traction force, active force, and quiescent force exerted by young and old VSMCs (*n* = 50). Active force is the force being exerted on micropillars that is three scaled median absolute deviations (MAD) away from the median force exerted on the pillars in the cell. (l) Traction force per pillar exerted by young and old VSMCs (*n* = 50). (m) Regression lines showing correlation between total traction force and cell area of young and old cells, each data point represents measurement of an individual VSMC. Straight lines denote the linear least‐squares fits to the data, and the shaded regions indicates the 90% confidence interval for each fit (*n* = 50). (n) Correlation between young and old VSMCs' steady‐state total traction force and peak force generated during instantaneous mechanoresponse (*n* = 8). In d, f‐h, and j‐n, data are represented as mean values ± SEM. *p* values were calculated using Student's *t* test, *p < 0.05, **p < 0.005, ***p < 0.0005, ****p < 0.00005.

The allostatic adaption VSMCs to a mechanical perturbation relies on the interaction of the CSK contractile components to generate cellular force, as well as the engagement of the mechanosensitive signaling elements, that is, the interplay among mechanosensitive ion channels, integrin‐focal adhesion‐actin axis, and calcium signal that convert the mechanical input into cellular contractile response (Hoffman et al., 2011). To define the mechanisms underlying aging‐associated decline in mechanosensation and VSMC functions, we performed transcriptome analysis of young and old VSMCs using DNA microarray analysis (Figure 2a). The transcriptome analysis revealed that 234 and 73 genes had significantly down‐ or up‐regulated expression in old versus young VSMCs, respectively (Figure 2b). To examine how the differentially expressed genes alter functional behaviors of VSMCs in aging, we clustered the differentially expressed genes into different pathways using Gene Ontology (GO) databases. The GO enrichment analysis indicated that several biological processes related to vascular functions were altered with aging (Figure 2c, colored blue; elaborated pathway analysis is included in Figure S7). Enrichment in inflammatory processes (colored red) and nuclear factor‐κB (NF‐κB) signaling suggest pro‐inflammatory state and cellular senescence that are often associated with aging (Ferrucci & Fabbri, 2018). Moreover, several pathways associated with vascular contractility and mechanosensory behaviors (colored green) were altered in aged VSMCs including vascular smooth muscle contraction, cell adhesion, cell‐extracellular matrix (ECM) crosstalk, focal adhesion assembly, regulation of the CSK, detection, and response to mechanical simulation (Figure 2c), supporting the maladaptive mechanosensation in old VSMCs measured by the ultrasound tweezers system.

To confirm the aging‐associated vascular dysfunctions, we profiled gene expression of previously described markers of VSMC functions and senescence, using quantitative reverse transcription PCR (qRT‐qPCR) (Figure 2d) (Yap et al., 2021). Previous studies have demonstrated increases in cellular senescence, or cell cycle arrest, and senescence‐associated inflammatory response with the progress of age (Lasry & Ben‐Neriah, 2015). The qRT‐PCR analyses showed that genes indicating senescent state such as p16, p21, and p53, as well as inflammation‐associated cytokines such as matrix metalloproteinase 3 (MMP3) and TNFα were upregulated in old VSMCs. Such changes contributed to VSMC phenotypic switch from a contractile phenotype to a synthetic phenotype and potentiate the risks of cardiovascular disease development (Beamish et al., 2010). For its significance in VSMC functional behaviors, we performed gene expression analysis on gene encoded for phenotypic plasticity in young and old VSMCs. Healthy VSMCs normally exhibit a differentiated contractile state defined by a high expression of smooth muscle‐specific contractile and CSK genes (Davis‐Dusenbery et al., 2011). However, we found that smooth muscle‐specific genes including ACTA2 (alpha‐smooth muscle actin 2 or α‐SMA2), MYH11 (smooth muscle myosin heavy chain 11), RAC1 (Rac Family Small GTPase 1 or Rac1), and TAGLN (Transgelin) were significantly downregulated in old VSMCs. Meanwhile, genes indicating synthetic state such as COL3A (Collagen Type III Alpha 1 Chain) and MMP9 (matrix metallopeptidase 9) were strongly upregulated in the old cells. Notably, a strong upregulation of matrix stiffening markers including COL1A (Collagen Type I Alpha 1 Chain) and COL6A (Collagen Type VI Alpha 1 Chain) and CSK stiffening markers ACTG2 (smooth muscle‐gamma actin 2 or γ‐actin2), ATCN2 (α‐actinin2), and were also observed in these aged cells. The altered gene expression patterns led us to interrogate CSK remodeling, aberrant mechanosensitive signaling, and contractile dysfunction as the culprit of defective mechanoresponse in old VSMCs.

We next assessed the expression and organization of CSK proteins at the single‐cell perspective. We investigated reorganization of the actomyosin CSK with immunostaining and an image recognition‐based spatial analysis to characterize partial deviation of the actin stress fibers (Figure 2e–h and Figure S8), in which partial deviation defines the actin alignment uniformity and reflects the bundling and organization of the actin filaments in VSMCs. (Y. Liu et al., 2018). Quantitative analysis of CSK architectures indicates that old VSMCs have disrupted actomyosin CSK with lower levels of actin filaments (F‐actin) and smooth muscle myosin heavy chain (SM‐MHC) (Figure 2e, f), along with a higher partial actin deviation (Figure 2h and Figure S8) relative to the young cells, suggesting that the local and total CSK filaments adapted a more diffused and disordered organization with the increase of age. We confirmed that the distinct differences in morphology and actin arrangement between the young and old cell groups were conserved both on the micropillar and flat glass substrates (Figure S9).

We further explored different smooth muscle‐specific actin isoforms and found that aging resulted in a reduction of α‐SMA and an upregulation of γ‐SMA (Figure 2e, f). Disruption in α‐SMA contractile filaments lead to dysfunction in CSK tension development in response to stimuli (Massett et al., 2020), and thereby, is responsible for a decline in VSMC mechanosensation. In addition, γ‐SMA in VSMCs resides mainly near the cell cortex and take part in force transmission along the actomyosin‐focal adhesion‐integrin axis (Kajuluri et al., 2021). An increased in γ‐SMA has been implied to be a contributor of increased cortical stiffness in previous studies (Kajuluri et al., 2021; Seawright et al., 2018). In aged cells, distribution of γ‐SMA stress fiber along the cytoplasm in the absence of α‐SMA promoted a stiffening CSK, as indicated by the lower ratio of α‐SMA (contractile element) to γ‐SMA (stiffening element) in old cells relative to the young cells (Figure 2g).

The measured aging‐associated structural alternations in CSK is consistent with the DNA microarray and qRT‐PCR analysis results, and suggested aging caused VSMCs transiting from a mechano‐active to a more inert mechanophenotype, resulting in an impairment in their mechanosensation to mechanical stress. We measured VSMC traction force at their passive, or unstimulated state, where aged VSMCs showed a significant increase in cell area (Figure 2j), exerted a higher total traction force (Figure 2k) but lower average force per pillar compared with that of the young cells, suggesting a shift to a more rigidified phenotype with aging (Figure 2j, k). Further quantitative analysis showed a positive correlation between the total traction force and cell area (Figure 2m). The regression lines were nonparallel, with the slope of the old cells' regression line being lower compared to the young cells, indicating distinct biomechanical properties between the young and old cells groups. Moreover, while old VSMCs generally have a higher traction force at the passive state, they showed compromised ability to generate mechanoallostasic force compared to the young cells (Figure 2n). This result supported the observations of impaired CSK integrity and inability of the old cells to generate force during allodynamic process upon mechanical stimulation.

### 2.3 Piezo1 regulates VSMC mechanosensation via Ca^2+^ signaling and dynamic cytoskeletal remodeling

**FIGURE 3:**
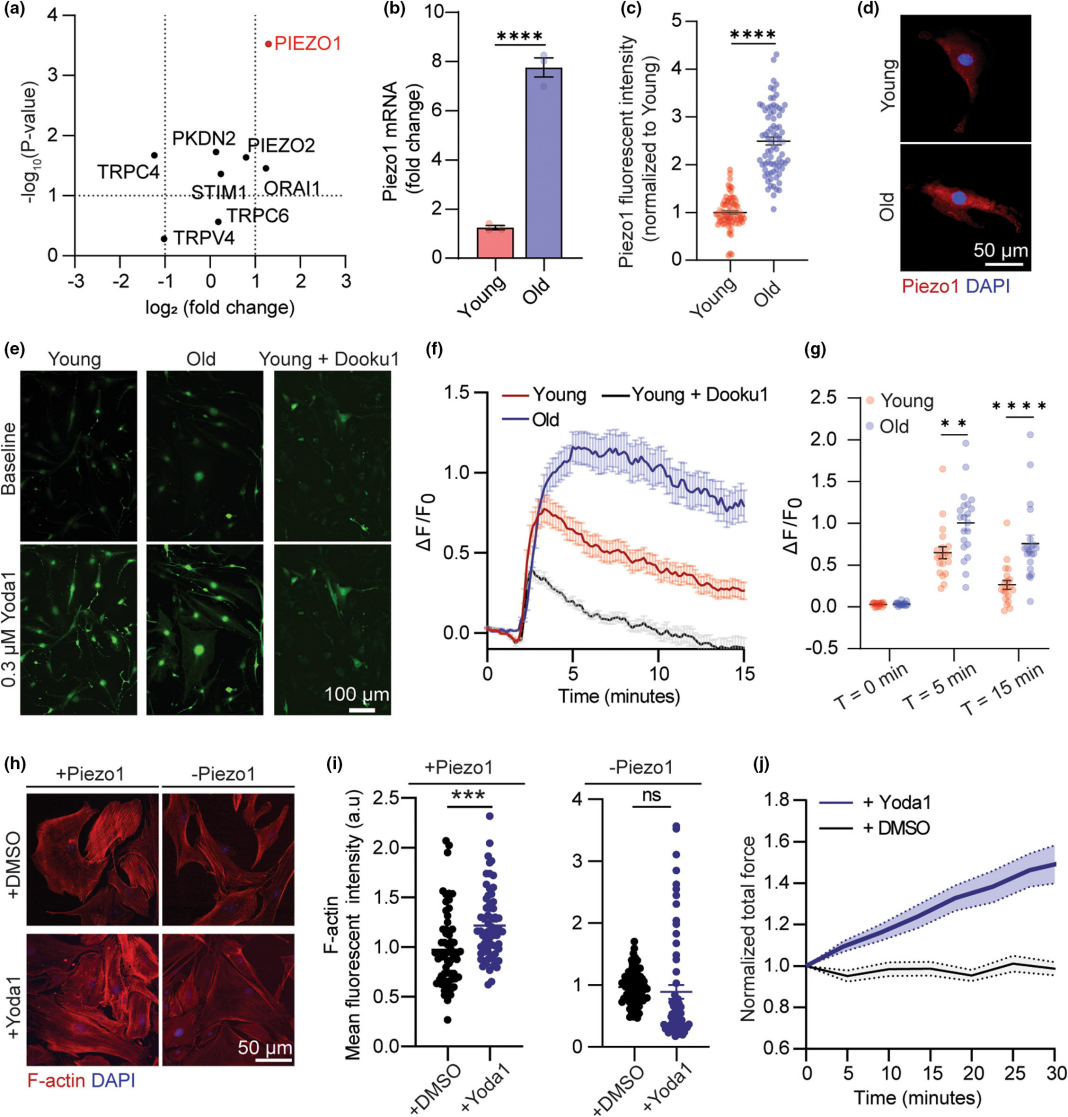
Piezo1 regulates VSMC mechanosensation via Ca^2+^ signaling. (a) Dot plot representation showing differential expression (log2) and *p* value of mechanosensitive channel transcripts in old verses young VSMCs. (b) qRT‐PCR (*n* = 4) and (c) immunofluorescent staining analysis of Piezo1 expression in young and old VSMCs (*n* = 50 cells per group). (d) Representative immunofluorescent images of Piezo1 in young and old VSMCs. (e) Representative images of cytosol Ca^2+^ signal in young, old, and young + Dooku1 cells transfected with Fluo4AM probe. (f) Time course measurement and (g) quantification of Ca^2+^ peak response in VSMCs treated as indicated in response to Yoda1 stimulation (*n* = 20, 20, 5 for young, old, and young+Dooku1 respectively, three independent experiments). Intensity of Fluo4AM was normalized to T = 0 min. (h) Representative immunofluorescent images of F‐Actin of young VSMCs under different treatment conditions. +DMSO and + Yoda1 represent VSMCs incubated with DMSO and Yoda1 for 1 h, respectively. +Piezo1 represents control group, and ‐Piezo1 represents inhibition of Piezo1 activation using Dooku1. (i) Quantification of F‐Actin (Phalloidin intensity) of VSMC in indicated groups (*n* = 50 cells per group). (j) Normalized traction force dynamics of young VSMCs in response to Yoda1‐mediated Piezo1 activation (*n* = 5). +DMSO represents control group. The thick line represents the mean of traction forces over time and the filled area represents the error bar. All statistical analysis was performed by Student's *t* test and error bars are presented as ± SEM. **p < 0.005, ***p < 0.001, ****p < 0.0005.

We next explored the upstream signaling pathway that mediates the decline in VSMC mechanosensation behaviors with aging. Mechanosensation depends on the cell‐ECM interaction that promotes conformational changes of the cell membrane and activates the mechanosensitive ion channels in response to mechanical stimuli (Mofrad et al., 2005). In VSMCs, calcium ions have been proven as the “second messengers” molecules that transduce external stimulation into the intracellular CSK components to trigger actomyosin contractility (Damm & Egli, 2014). Therefore, our next step was to profile gene expression of the common calcium‐regulating ion channels (S. Liu & Lin, 2022) in VSMCs using qRT‐PCR to identify the key mediator of VSMC dynamic mechanoresponse. We found that Piezo1, a mechanosensitive channel with preference to changes in Ca^2+^ concentration, was remarkably upregulated in old VSMCs (Figure 3a). We performed qRT‐PCR analyses of Piezo1 gene expression in young and old VSCMs isolated from five different mice and confirmed that Piezo1 expression increased by 2–5 folds in old VSMCs relative to the young cells (Figure 3b and Figure S10). The qRT‐PCR results were further confirmed by immunostaining at protein levels (Figure 3c,d). The Piezo1 ion channel has been known as a key mechanical sensor on the cell membrane regulating Ca^2+^ influx upon its activation driven by external mechanical force and influence cellular force generation since actomyosin contraction is initiated by calcium influx (Chen et al., 2018). The role of Piezo1 has been previously discussed in cardiovascular disease interventions, including vascular development, blood pressure, hypertension‐dependent arterial remodeling (Retailleau et al., 2015), and VSMCs with aneurysm (Qian et al., 2022), but it remains unexplored in chronological aging. We suspected that the hyperactivation of Piezo1 and Piezo1‐mediated calcium signaling are the key mediators of maladaptive mechanosensory behaviors of aged VSMCs.

To confirm whether Piezo1 activation determines Ca^2+^ signaling, we transfected the VSMCs with Fluo4 AM, a calcium indicator and quantitatively evaluate changes in cytosolic calcium in response to activation of Piezo1 by Yoda1—a Piezo1 chemical agonist (Botello‐Smith et al., 2019). Upon addition of 0.3 μM Yoda1, there was an immediate elevation in Ca^2+^ intensity in both young and old VSMCs, followed by a gradual decrease to reach internal homeostasis (Figure 3e,f). Calcium signals were significantly suppressed in the presence of Dooku1, a Yoda1 antagonist that prevents Piezo1 activation, confirming the specificity of calcium influx in VSMCs to Piezo1 channel (Figure 3f). The old cells exhibited a higher peak calcium response and a more sustained calcium signal explained by a more abundant presence of Piezo1 compared to the young cells (Figure 3f). After the 15 min observation, calcium signal in the young cells were able to mostly return to the baseline level, while the calcium concentration in the old cells only decreased by 25% (Figure 3g). Altogether, quantification of calcium signaling reinforces that the level of Piezo1 expression determines the persistence of calcium signaling in VSMCs. This conclusion is supported by Pan's et al findings that Piezo1 triggers a biphasic response of intracellular calcium, where overexpression of Piezo1 causes a prolonged calcium signaling and disruption in the actomyosin‐focal adhesion‐ECM pathway (Pan et al., 2022). This calcium patterns reflected the biphasic mode of VSMC allostasis force dynamic measured by the ultrasound tweezers system (Figure 1g), suggesting that Piezo1‐dependent calcium signaling regulates the persistence of the mechanoresponse in VSMCs, while the activation level of contraction is dependent on the concentration of contractile filaments presented in the CSK.

Ca^2+^ influx is an upstream signal that initiates tension development in the CSK via cooperation of the actomyosin complex (Hartshorne & Mrwa, 1982). The natural next step we took was to interrogate Piezo1 as the long‐range mediator for actin remodeling and force generation in young (healthy) VSMCs. We performed quantitative assessment of F‐actin intensity after 1 h of Yoda1 treatment using phalloidin staining. Yoda1‐induced Piezo1 activation resulted in an elevation of F‐actin expression compared to their basal state (Figure 3h, i). Conversely, no significant change was detected in cells treated with Dooku1 in prior to treatment of Yoda1. These findings revealed that opening of Piezo1 mechanosensitive channel is essential for actin polymerization in response to extracellular stimuli. Next, we measured Yoda1‐stimulated changes in CSK tension of VSMC using the micropillar array and confirmed that Piezo1 activation led to an increase in force response of VSMCs (Figure 3j). Altogether, our results identified Piezo1 as a critical regulator of calcium signaling and CSK force development in VSMCs and suggested that overexpression of Piezo1 is the upstream culprit of the maladaptive mechanoresponse in old VSMCs.

### 2.4 Piezo1 expression can be fine‐tuned by in vitro aging models

Based on the experimental results above, we asked whether extenuating Piezo1 overexpression would mitigate the loss in mechanosensitivity of the old cells. We therefore established two in vitro models that aimed to fine‐tune Piezo1 expression to regulate mechanical aging of VSMCs. In the first model coined “Piezo1‐reversed aging”, we performed siRNA transfection with ON‐TARGETplus siPiezo1 at 25 nM for 48 h to reduce Piezo1 at mRNA level in old VSMCs. The second model called “induced premature aging” involves acceleration of aging in young VSMCs by pharmacological treatment of 10^−7^ M Angiotensin II (AngII) for 48 h. AngII has been found to provoke vasoconstriction, inflammation, and vascular remodeling, thus lead to cardiovascular disease development, such as atherosclerosis and hypertension (Wu et al., 2021). Kunieda et al discovered that AngII‐induced premature cellular senescence of human VSMCs via a p21‐dependent pathway (Kunieda et al., 2006). Our gene expression analysis revealed a strong correlation between aging and cellular senescence, where old cells showed upregulation of cell cycle proteins, notably p21, (Figure 2d). Thus, cell senescence‐driven aging by AngII would be an efficient in vitro model to mimic the effects of chronological aging on VSMC Piezo1‐dependent mechanosensation.

**FIGURE 4:**
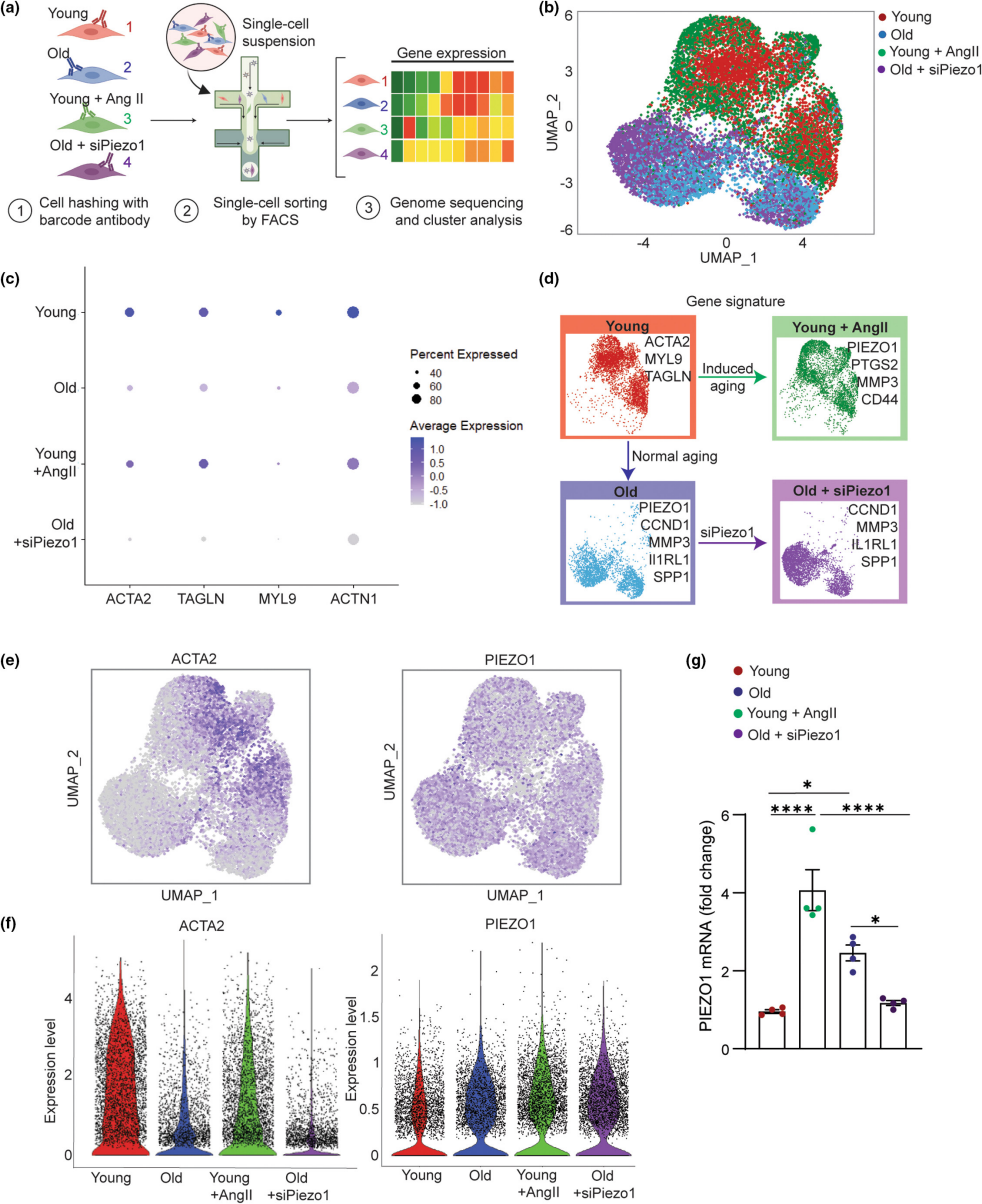
Single‐cell sequencing analysis of in vitro VSMC aging models. (a) Schematic showing study overview. (b) UMAP (uniform manifold approximation and projection) visualization of color‐coded clustering of the VSMC in the four experimental groups (young, young + AngII, old, and old + siPiezo1). (c) Dot plot showing average and percent expression of genes encoded for vascular cell markers in the four cell clusters. (d) Summary showing upregulated genes in each cell cluster. (e) Representative UMAP showing expression levels of smooth muscle α‐Actin (ACTA2) and Piezo1 in the four cell clusters. (f) Violin plots comparing expression level of ACTA2 and Piezo1 in four experimental groups. (g) qRT‐PCR analysis of Piezo1 expression in VSMCs of young, young + AngII, old, and old + siPiezo1, respectively (*n* = 4). All data are represented as mean values ± SEM. *p* values were calculated using one‐way ANOVA followed by Tukey's post hoc test, *p < 0.05, ****p < 0.00005. Figure 4a is created with BioRender.com.

To elaborate on VSMC heterogeneity and functions among the regulated aging pathways, we performed transcriptome profiling using single‐cell RNA sequencing (scRNA‐seq). Single‐cell suspensions were prepared and sequenced for AngII‐treated (induced premature aging) young cells and the siPiezo1‐treated (Piezo1‐reversed aging) old cells, as well as the control young and old cells (Figure 4a). The clustering analysis captured three distinctive regions among the sequenced cells. Based on the experimental conditions of the input, we were able to distinguish the young, old, and AngII‐treated young cell, and siPiezo1‐treated old cells groups (Figure 4b, Figure S11A,B and Table S1). The young cells were characterized by abundant expression of VSMC markers, such as ACTA2 and MYL9 (myosin light chain 9), TAGLN while the old and AngII‐treated young cell groups were classified by a significant upregulation of aging‐associated genes. Particularly, we found that genes related to cellular senescence (e.g., CCND1, CCND2, ETS1, PTGS2, and ILRL1) and senescence‐associated secretory phenotype (MMP3) were highly concentrated in the clusters of old and AngII‐treated young cells (Figure S11C,E). The enriched expression of MMP3 in VSMCs were previously shown to be regulated by Piezo1 activation in VSMC of aortic aneurysm, suggesting that the old and AngII‐treated young cells are associated with dysfunction in mechanosensory behaviors and high risk of cardiovascular disease development (Qian et al., 2022). Notably, we found that the AngII‐treated young cell cluster showed the highest amount of CD44 (macrophage‐like VSMC marker) among the four groups, while SPP1 (secreted Phosphoprotein 1, osteogenic VSMC marker) was only found in the old and siPiezo1‐treated old cell groups (Figure S12). Based on previous reports of the multidirectional phenotypic switch in VSMCs in response to different extrinsic signal, we hypothesized that VSMC transformed into osteogenic state during chronological aging and macrophage‐like state in AngII‐induced premature aging (Cao et al., 2022). Interestingly, despite the considerable difference between the AngII‐treated young cell and the old cell clusters, we found a resemblance in the differential expression pattern of the CSK markers between the old and AngII‐induced premature aging groups. By profiling the contractile genes such as ACTA2, ACTG1 (γ‐actin2), TAGLN, and ACTN1 (α‐actinin1), we confirmed that AngII treatment is efficient in mimicking the effect of vascular aging on VSMC CSK integrity. The single‐cell gene profile between young and young AngII group showed a downregulation of α‐actin, in contrast to an upregulation of γ‐actin, transgelin, and α‐actinin (Figure 4c, e, f). The results resembled our qRT‐PCR and immunostaining results measuring changes in contractile gene expression with normal aging, as shown in Figure 2d–f. Thus, it is suggested that pharmacological treatment of young cells with AngII disrupted the CSK‐mediated contractile properties in young cells and promoted their transition into a more mechanically passive state, while siPiezo1 treatment does not alter the CSK integrity and the baseline mechanical behaviors of old cells.

Next, we examined the mechanosensing receptors presented in the four cell clusters (Figure S11C). Among the mechanosensitive channels, Piezo1 was highly expressed and was positively associated with aging pathway, indicated in both old and AngII‐induced premature aging groups (Figure 4e,f). Importantly, siPiezo1 treatment significantly reduced the Piezo1 expression level in the old cells, confirming successful siRNA transfection (Figure S11C). We further validated the results using qRT‐PCR and found that Piezo1 mRNA level was alleviated by 1.5 folds in the siPiezo1‐treated old cells compared to the old cells, while there is no significant difference between the siPiezo1‐treated old cells and young cells. This result suggested that overexpression of Piezo1 is the hallmark of aging and aging‐associated cardiovascular disease in VSMCs. From the single‐cell analysis of upregulated genes in each experimental cluster, we confirmed that while AngII treatment induced vascular aging in a different pathway compared to chronological aging, it provided a considerably efficient method to regulate aging in vitro by mimicking the changes in contractile functions and mechanosensitive Piezo1 channels. Meanwhile, siPiezo1 transfection is good for specific targeting of Piezo1 expression to fine‐tune the mechanosensitive signaling pathway without shifting the phenotypic signature of the old VSMCs (Figure 4g).

### 2.5 Antagonizing Piezo1 mitigates aging‐mediated aberrant mechanosensation in old VSMCs

**FIGURE 5:**
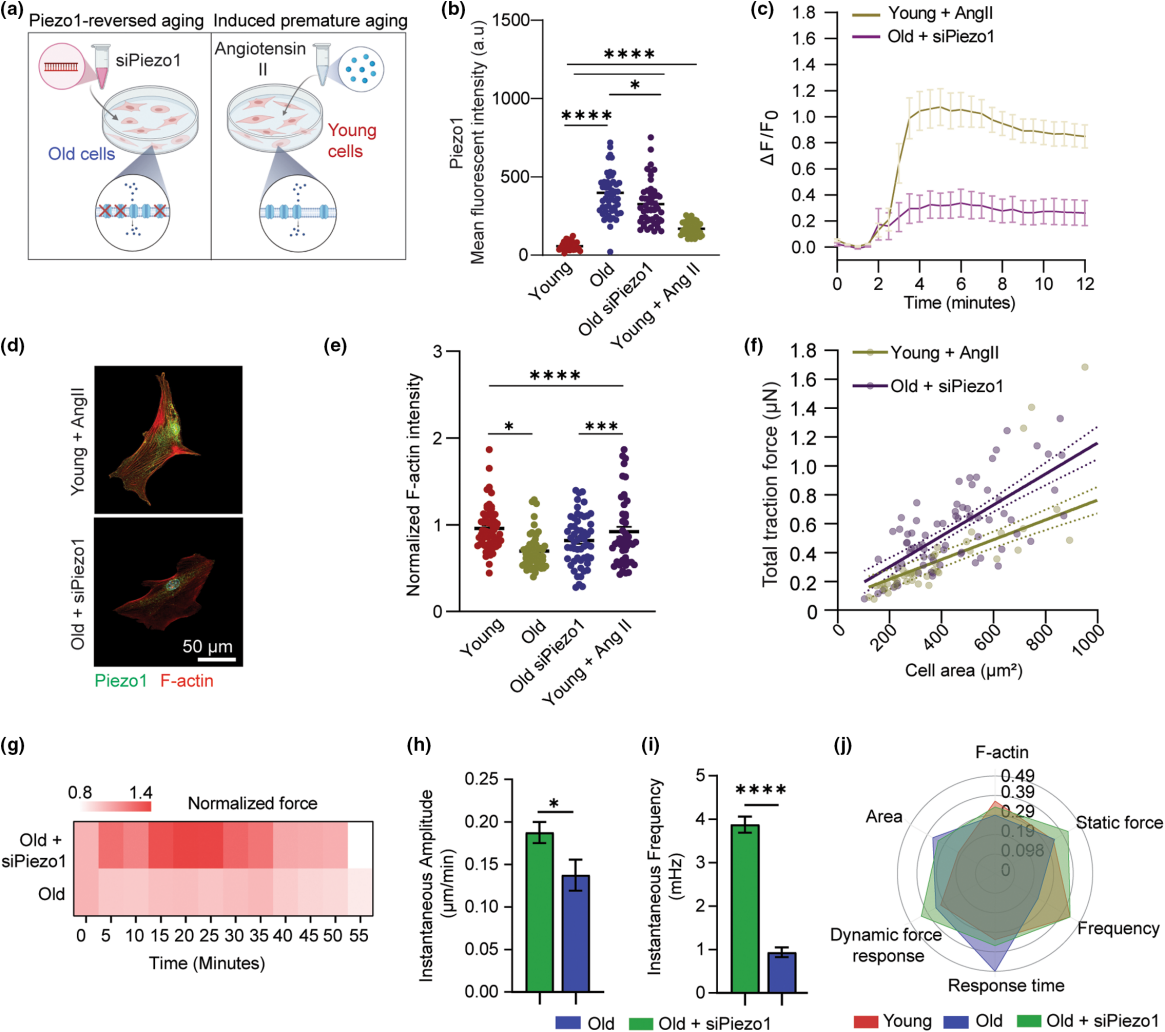
Fine‐tuning Piezo1 recovered mechanosensation in old VSMCs. (a) Schematic showing two in vitro models using pharmacological treatments to mimic biological aging and regulate Piezo1 expression. (b) Immunofluorescent staining analysis of Piezo1 expression in VSMCs of young, young + AngII, old, and old + AngII respectively (*n* = 50). (c) Time course of Ca^2+^ signal in VSMCs treated as indicated in response to Yoda1 stimulation (*n* = 20 cells per group, three independent experiments). Intensity of Fluo4AM was normalized to T = 0 min. (d) Representative immunofluorescent staining images of Piezo1 and F‐Actin fluorescent expression in young + AngII and old + siPiezo1 VSMCs. (e) Quantification of changes in F‐Actin expression (Phalloidin) in young versus young + AngII VSMCs and old versus old + siPiezo1 VSMCs (*n* = 50 cells per group). Fluorescent intensity of young versus young + AngII group was normalized to young, and fluorescent intensity in old versus old + siPiezo1 group was normalized to old cells. (f) Regression lines showing correlation between total traction force and cell area of young + AngII and old + siPiezo1 cells. Each data point represents measurement of an individual VSMC. Straight lines denote the linear least‐squares fits to the data, and the shaded regions indicates the 90% confidence interval for each fit (*n* = 50). The data are normalized to the average cell area and average traction force of the young cells. (g) Temporal heatmap of force response of old cells treated with siPiezo1 and old cells upon ultrasound stimulation (*n* = 8), results are normalized to average force response of old cells. (h) Instantaneous amplitude and (i) Instantaneous frequency of old versus old‐siPiezo1 cells (*n* = 8). (j) Representative radar chart showing the average values of mechanical phenotypes of VSMCs in different groups. Data are normalized between 0 and 1 using min‐max normalization. In b, c, e, h, and i, data are represented as mean values ± SEM. In h and i, statistical analysis was performed by Student's *t* test. In b and e, statistical analysis among the four groups was performed by ANOVA followed by Tukey's post hoc test. *denotes *p* < 0.05, ***p < 0.001, ****p < 0.00005. Figure 5a is created with BioRender.com.

To further evaluate Piezo1 mechanosensitive signaling in the established in vitro models (Figure 5a), we assessed Piezo1‐mediated calcium signaling and mechanoresponse of VSMCs in each group upon Piezo1 activation. We first performed immunostaining to detect the protein expression level of Piezo1 and found that Piezo1 expression was alleviated 1.8 folds at protein levels in the “Piezo1‐reversed aging” cells compared to the old cells (treated with nontargeting siRNA). In the AngII‐induced “premature aging” model, Ang II‐treated young cells showed an increase in Piezo1 by 2 folds compared to the control young cells (Figure 5b). We next examined whether manipulating Piezo1 expression in the two designed cell groups can mimic the calcium signaling patterns that we observed in VSMCs during chronological aging by applying Yoda1 chemical stimulation. In response to Yoda1 addition, both the siPiezo1‐treated (Piezo1‐reversed aging) old cells and the AngII‐induced premature aging cells exhibited a rise in cytosolic Ca^2+^ compared to their basal state, with a higher calcium peak observed in the AngII‐treated cells. After 10‐min treatment of Yoda1, the Piezo1‐reversed aging cells were able to achieve a homeostasis condition, while the AngII‐induced premature aging cells fails to return to its baseline calcium level (Figure 5c). The results not only validated the efficiency of pharmacological treatments to simulate the mechanical aging process in vitro, but also confirmed the regulatory roles of Piezo1 on calcium signaling in VSMCs. Furthermore, silencing Piezo1 prompted the promising approach to enervate the hyperactivation of Piezo1 in old cells while ameliorated their ability to adapt rapidly to an external stimulation in calcium‐related niche. Next, we investigated actin CSK and static force of VSMCs in the Piezo1‐reversed aging and AngII‐induced premature aging models. Inhibition of Piezo1 in old cells had no significant effects on their F‐actin intensity, implicating that Piezo1 plays a less important role on the CSK at the nondynamic level (Figure 5d,e). Meanwhile, AngII treatment resulted in a decrease by 2.4 folds in F‐actin concentration in young cells, therefore perturbing their ability to generate dynamic force in response to a mechanical stimulation. Overall, the AngII‐induced premature aging group using AngII treatment demonstrated a total shift in the mechanophenotype of young VSMCs, including changes in actin CSK, cell area, and static traction force while the Piezo1‐reversed aging group using siPiezo1 treatment conserve the biophysical properties of the old cells at the baseline perspective (Figure 5f).

Building upon these findings, we next measured whether reducing Piezo1 overexpression aid in recovery of VSMC mechanosensation by applying the ultrasound tweezers system measurement to probe force response of old cells treated with siPiezo1. Upon a 10‐s transient stimulation, we observed that the old cells treated with siPiezo1 exhibited a higher mechanosensitivity, as demonstrated by a stronger increase in cellular force compared to the control old cells (Figure 5g). The treated cells also recovered their biphasic mechano‐allostasis behavior with an excitation period from 15 min to 37.5 min, followed by a gradual decrease in force for the remaining 17.5 min. To validate the effect of siPiezo1 treatment on reversed mechanical response, we conducted siRNA transfection with a different siRNA (Life Technologies) targeted against Piezo1 and found similar Ca^2+^ response and restore in force response of siPiezo1‐treated old cells comparing to the controls (Figure S13). We then applied spectral analysis to examine the instantaneous characteristics of the two cells group through their temporal force response. We found that the old cells treated with siPiezo1 showed a significantly higher instantaneous amplitude compared to the control old cells during the 55‐min period (Figure 5h), and a higher instantaneous frequency distribution compared to the old cells with no treatment (Figure 5i). This shift in instantaneous frequency spectra indicated that inhibiting overexpression of Piezo1 in old cells improved their force‐generating capacity as well as the dysfunctions in mechanosensitive signaling, thereby restored their ability to adapt rapidly to external mechanical stimulation. Overall, antagonizing Piezo1 expression considerably restored the old cells' mechanosensing properties to resemble that of the young cells (Figure 5j). Ours results highlighted the tremendous efficiency of fine‐tuning Piezo1 in regulating the mechanosensitive signaling pathway underlying VSMC mechanoresponse and suggested that suppressing Piezo1 expression would provide a therapeutic treatment to partially ameliorate the biophysical loss caused by aging in VSMCs.

## 3 DISCUSSION

The dynamic interplay between the mechanosensitive receptors and the intracellular structures allows VSMCs to alters their physiological and biophysical behaviors to rapidly adapt to the mechanical perturbations in the microenvironment and maintain an internal homeostasis. Aging leads to detrimental disruptions in VSMCs' mechanosensitive behaviors from the cellular and subcellular levels. Using the ultrasound tweezers system, we explored the compromised mechanosensation regarding a decline in contractile force and a delayed force response time in old VSMCs after exposure to a transient mechanical stimulation. Such a unique ultrasound tweezers system that enables manipulation over the mechanical force applied to single cells, in situ assessment of cellular force dynamics (e.g., response time and contractile magnitude), and subsequent advanced signal processing (e.g., instantaneous force frequency analysis) for in‐depth analysis of VSMC's intrinsic mechanosensation behaviors. Our previous study on VSMCs with progressive aortic aneurysm also revealed a blunt mechanosensation in VSMCs, yet the force response pattern to mechanical stimulation of old VSMCs in chronological aging is somewhat different from those with the pathological condition such as aneurysm, where delay in force response was observed in VSMCs with chronological aging compared to healthy and aneurysm cells (Qian et al., 2022).

From DNA microarray analysis, we found that old VSMCs were strongly correlated with cellular senescence and inflammatory responses, as well as significant alterations in the pathways associated with vascular functions and mechanosensation. Aging also causes a phenotypic switch of VSMCs from a contractile phenotype to a synthetic phenotype, leading to defective contractile functions and stiffening of the CSK. Specifically, we found a significant alleviation of contractile markers in old VSMCs (e.g., F‐actin, α‐actin, and myosin) and an upregulation of CSK stiffening markers (γ‐actin). Notably, other studies by Massett et al and Seawright et al also emphasized the increased of γ‐actin throughout in the loss of α‐actin as the subcellular culprit behind the decline in contractile functions and mechanosensing in VSMCs (Massett et al., 2020; Seawright et al., 2018). Our study defines the ratio of α‐actin over γ‐actin as a complementary “stiffening index” to characterize the alternations in CSK integrity and VSMC mechanosensation with aging.

This study uncovered the role of Piezo1 mechanosensitive signaling in aging‐dependent mechanobiological dysfunctions (Figure S14). We first discovered a substantial elevation in Piezo1 expression in old VSMCs, which was a pathological hallmark indicated in previous studies on vascular cells with pulmonary arterial hypertension (Liao et al., 2021) and cardiomyopathy (Jiang et al., 2021). Our study revealed that the overexpression of Piezo1 in old VSMCs mediated abnormal Ca^2+^ signaling and altered their downstream mechanosensitive pathway. Activation of Piezo1 and increased intracellular Ca^2+^ initiates the sliding of actomyosin complexes, resulting in an increase in cellular force (Nourse & Pathak, 2017). In normal young cells, Ca^2+^ influx is transient upon Piezo1 activation, while in the old cells, increased Ca^2+^ is sustained for a prolonged period. It suggests that the rise in intracellular Ca^2+^ in old cells exceed the physiological requirement within VSMCs, thereby inducing dysfunctions in vascular mechanosensation.

Our study further provided a potential “mechanomedicine” strategy aiming to reduce Piezo1 overactivation in aged VSMC to restore a healthy mechanosensation, and thus alleviate the mechanical disruptions in vascular aging. We designed two models utilizing pharmacological treatments to induce “premature aging” using AngII and “Piezo1‐reversed aging” by antagonizing Piezo1 expression with siRNA transfection. The gene signature in each gene cluster indicated that AngII‐induced premature aging in young cells can partially mimic in vivo chronological aging. Meanwhile, siRNA transfection was efficient to antagonize Piezo1 overexpression without altering the transcriptomic signature of the old VSMCs. We confirmed that AngII‐treated young cells exhibited an impairment in Ca^2+^ signaling, thus induced dysfunctional mechanosensitive signaling that was found in the normal old cells. Conversely, reducing Piezo1 overexpression in the old cells by siRNA transfection ameliorates their mechanosensation, confirming by a recovery in instantaneous frequency response upon ultrasound tweezers stimulation. Yet, the in vitro AngII‐induced premature model may not best mimic the chronological aging process, thus it is crucial to validate the findings of this study in the future with in vivo experiments. Piezo1 knock out mice and biaxial testing could be used to validate the role of Piezo1 in mediating vasoconstriction response of VSMCs in aging. Building upon this finding, further studies could be done to examine how Piezo1 gain‐of‐function mutation could potentially lead to accelerated failures in the function of vascular cells and early vascular aging in different geographical populations.

Taken together, combining the ultrasound tweezers system, transcriptome profiling, and advanced mechanobiological analytic methods, we established a complete framework for the analysis of VSMC mechanosensation behaviors and characterizations of the mechanosensation dysfunction associated with aging. Our study reveals that abnormal expression of mechanosensitive Piezo1 ion channel plays a long‐range regulatory role in aging‐associated impairment in VSMC mechanosensation. Additionally, our established framework opens a world of possibilities for broader applications, that are not only limited to vascular cells, but to any adherent cell, and across a multitude of problem areas such as determining etiology of diseases like cardiovascular diseases, diabetes, Alzheimer's, and many more.

## 4 METHODS

### 4.1 Cell culture

Young and old mouse primary aortic VSMCs (CSC‐C4696L and CSC‐C4301X from Creative Bioarrays, C57‐6080 and A57‐6080 from Cell Biologics) were isolated from aortas of C57BL/6 mice of 12 weeks and 58–78 weeks. CSC‐C4696L and CSC‐C4301X VSMCs were cultured in SuperCult® Mouse Aortic Smooth Muscle Cell Medium Kit (Creative Bioarrays), C57‐6080 and A57‐6080 VSMCs were cultured in Complete Smooth Muscle Cell Medium (Cell Biologics). For all experiments, primary VSMCs were cultured and used for no more than three passages.

### 4.2 Micropillar array fabrication and functionalization

The PDMS micropillar array was fabricated using a standard soft lithography two‐step molding process (details see Appendix S1) (Yang et al., 2011). Fabricated PDMS micropillar array substrates were mounted on a 60 mm petri dish with a 15 mm hole in the center. PDMS micropillar arrays were functionalized using microcontact printing with fibronectin (50 μg/mL; Sigma‐Aldrich) and Alexa‐Fluor 647‐conjugated fibrinogen (25 μg/mL; Life Technologies). VSMCs were then seeded on the functionalized PDMS micropillar array in the petri dish and cultured overnight prior to experimentation. Schematic illustration of micropillar fabrication and functionalization is presented in Figure S2.

### 4.3 Mechanical stimulation of VSMCs using ultrasound tweezers

Biotinylated VesselVue microbubbles (Sonovol) of diameter between 4 and 5 μm were attached to VSMCs following a previous established protocol (details see Appendix S1) (Qian et al., 2022). VSMCs with single microbubbles attached were selected for experimentation with ultrasound tweezers. A 10‐MHz ultrasound transducer (V312‐SM, Olympus) was used to apply acoustic ultrasound pulses to move the microbubbles so as to stretch the cell membrane at a frequency of 1 Hz for a period of 10 s. Before experiments, ultrasound transducer was aligned using a pulser receiver (Olympus) to fix at a 45° angle and a 11.25 mm distance (Rayleigh distance) from the target cell under an inverted microscope (Zeiss Axio Observer Z1). A function generator (Agilent Technologies 33,250 A) along with a 75‐W power amplifier (Amplifier Research 75A250) was used to drive the ultrasound transducer.

### 4.4 Quantification of traction force and strain energy

After the ultrasound stimulation, deflections of individual fluorescence‐tagged micropillars underneath cells were recorded continuously for 1 h with a 30‐s interval with an inverted microscope (Zeiss Axio Observer Z1). Traction force exerted by VSMC at each time point was quantified based on the displacements of the micropillars using Cellogram and customized MATLAB programs (Mathworks) (details see Figure S3) as described previously (Lendenmann et al., 2019; Qian et al., 2022). The magnitude of cellular strain energy is equal to the micropillar's deforming energy:

$$E_{\text{strain}}=\frac{1}{2}\sum_{i=1}^{N}\overset{⃑}{T_{i}}\left(x,y\right).\overset{⃑}{u_{i}}\left(x,y\right)$$

where $\overset{⃑}{T_{i}}\left(x,y\right)$ the traction force exerted by the cell at position (x,y) and $\overset{⃑}{u_{i}}\left(x,y\right)$ the displacement of micropillar at the position (x,y).

### 4.5 Spectrum analysis of VSMC force dynamics

The instantaneous force frequency spectrum analysis was obtained by tracking the time series of displacement velocity of individual micropillars following the ultrasound stimulation. Specifically, EMD was performed separates the velocity time series into a finite and small number of IMFs (a portion of the complete signal) to reduce noise in the original signal. HHT was then applied to each IMF to derive instantaneous frequency F(t) and instantaneous amplitude A(t) at each time point (t) (Ma et al., 2018; Qian et al., 2022), and generate the instantaneous spectrum that provides frequency magnitude and distribution of the input force dynamics data. In this study, IMF2 was used to calculate the instantaneous characteristics of VSMC force response due to its optimal noise reduction and resemblance with the original signal.

### 4.6 Immunofluorescence staining and microscopy

VSMCs were fixed in a 4% Paraformaldehyde (Alfa Aesar) solution and permeabilized with 0.2% Triton X‐100 (Roche Applied Science) in PBS (Gibco), and blocked with 3% bovine serum albumin (BSA, Sigma Aldrich) at room temperature. Staining was performed by incubating the cells overnight at 4°C with primary antibodies (1:100 diluted in PBS, Table S2), multiple washing with PBS with Tween 20 (PBST, ThermoScientific), and incubating diluted secondary antibodies and DAPI in 1% BSA for 1 h at room temperature. After staining, immunofluorescence images were captured using a Zeiss LSM 710 confocal microscope (Carl Zeiss).

### 4.7 Calcium imaging

VSMCs were washed twice using Tyrode solution (Electron Microscopy Sciences), then transfected with calcium indicator Fluo‐4 (Thermo Fisher Scientific) for 30 min at room temperature and washed with Tyrode solution for three times. Imaging of calcium influx were captured using an inverted microscope (Zeiss Axio Observer Z1) at 480 nm excitation wavelength for 30 min at 10‐s interval. Yoda1 (0.3 μM, Cayman Chemical) was added 2 min after starting recording.

### 4.8 qRT‐PCR analysis

RNA isolation was performed using the Directzol RNA MiniPrep Kit (Zymo Research). Following reverse transcription using the cDNA Synthesis Kit (Bio‐Rad), quantitative real‐time PCR was performed with Fast SYBR Green Master Mix (Life Technologies) using a CFX96 Touch Real‐Time PCR Detection System (BioRad). The primer sequence set used were listed in Table S3.

### 4.9 DNA microarray analysis

RNA was isolated using RNeasy Mini Kit (Qiagen). Reverse transcription was performed based on manufacturer's recommendation for Affymetrix WT PLUS Kit. cDNA was then amplified and labeled using a Biotin Allonamide Triphosphate. The labeled cDNA was then cleaned up, fragmented, and hybridized to arrays for 16 h at 45°C. After hybridization, the probe array was washed on the Fluidics Station 450/250 following the instructions, and was scanned using the GeneChip® Scanner 3000. The oligo package (open source) was used to preprocess microarray data using R. Oligo utilizes RMA algorithm for background correct, quantile normalization and analysis of gene expression levels. Then the limma package was used for differential expression analysis. Differentially expressed genes were identified based on logFC>1, *p* value <0.05.

### 4.10 Piezo1 siRNA transfection

Knockdown of Piezo1 was performed by siRNA transfection with 20 nM of ON‐TARGETplus Piezo1 siRNA Reagents (L‐061455‐00‐0005, Horizon Discovery), ON‐TARGETplus nontargeting control pool (D‐001810‐10‐05, Horizon Discovery) or Silencer Pre‐designed Piezo1 siRNA (AM16708, Life Technologies). VSMCs were cultured in a six‐well culture plate at density of 250,000 cells per well overnight, following by addition of Lipofectamine™ 3000 (Invitrogen) with siRNA reagents according to the manufacturer's instruction and incubation for 48 h. The efficiency of Piezo1 knockdown was validated by immunofluorescence staining and qRT‐PCR.

### 4.11 scRNA‐seq analysis

VSMCs were oligo‐tagged with antimouse antibodies (TotalSeq A, Biolegend). The cell suspension was loaded on a 10x Genomics Chromium instrument. The libraries were prepared using Chromium Single Cell 3′ Library & Gel Bead Kit v3.1, PN‐1000268, the Chromium Next GEM Chip G Single Cell Kit PN‐ 1000120 and the Dual Index Kit TT, Set A PN‐1000215, (10x Genomics). Amplified cDNA was evaluated on an Agilent BioAnalyzer 2100 using a High Sensitivity DNA Kit (Agilent Technologies) and final libraries on an Agilent TapeStation 4200 using High Sensitivity D1000 ScreenTape (Agilent Technologies). Individual libraries were diluted to 2 nM and pooled for sequencing. Pools were sequenced with 100 cycle run kits (28 bp Read1, 8 bp Index1, and 91 bp Read2) on the NovaSeq 6000 Sequencing System (Illumina). Seurat package was used for quality control, data filtering, dimensionality reduction, differential gene expression analysis, and uniform manifold approximation.

### 4.12 Statistics and reproducibility

All data were from at least three independent experiments. Data were first analyzed for normality and then compared with two‐sided unpaired *t* test using Prism 8 software (GraphPad software Inc.). Multiple groups were compared by one‐way analysis of variance (ANOVA) followed by Tukey's post hoc test as indicated in the figure captions. *p* value smaller than 0.05 was considered statistically significant and sample sizes and exact *p* values are indicated in all figure captions.

## AUTHOR CONTRIBUTIONS

N. Luu, A. Bajpai, W. Chen designed study and wrote the manuscript; N. Luu, A. Bajpai R. Li, S. Park, and M. Noor performed the experiments and analyzed data; N. Luu, A. Bajpai, X. Ma developed the methodology; W. Chen supervised the study.

## CONFLICT OF INTEREST STATEMENT

The authors declare no conflict of interest.

## Supporting information


Appendix S1

Video S1

## Data Availability

The DNA microarray and scRNA‐seq datasets are available at Gene Expression Omnibus (GEO) under accession number GSE243736, GSE24411, and GSE230805. All other data that support the findings of this study are available from the authors upon request.
