# Supplementary material for: Aging‐associated decline in vascular smooth muscle cell mechanosensation is mediated by Piezo1 channel
Source: Aging Cell. 2023 Nov 9;23(2):e14036. doi: 10.1111/acel.14036 (PMC10861209; doi:10.1111/acel.14036)
Supplement: Supplementary file 1 — Appendix S1 [file ACEL-23-e14036-s002.pdf]

Supporting Information for

Aging-associated Decline in Vascular Smooth Muscle Cell Mechanosensation is Mediated by Piezo1 Channel

This Supporting Information file includes:

Supporting Methods.

Supporting Figures S1-S15.

Supporting Table S1-S3.

1. Supporting Methods.

Micropillar array fabrication

The PDMS micropillar array was fabricated using a standard soft lithography two-step molding process (Yang, Fu, Wang, Desai, & Chen, 2011). A silicon master mold (micropillar height 7.1 µm and diameter 1.8 µm) was fabricated using photolithography and deep reactive ion etching. Silanization of silicon molds was carried out with tridecafluoro-1,1,2,2, -tetrahydrooctyl)-1-trichlorosilane (Sigma-Aldrich) overnight in vacuum. Negative PDMS molds were made from the silicon master mold by adding Sylgard 184 silicone elastomer base and curing agent (Dow Corning) mixture by 10: 1 mass ratio to the silicon master mold and baking at 110°C for 30 mins. After curing, negative PDMS molds were peeled off from the silicon mold and silanizied in vacuum overnight. PDMS micropillar arrays were generated by casting a layer of Sylgard 184 silicone elastomer base and curing agent mixture by 10: 1 mass ratio on the surface of the silanized negative molds, and the negative molds were then covered with oxygen plasma (350 W, PlasmaEtch) treated glass coverslips (22mm x 22mm, Electron Microscopy). After curing in an oven heated at 110 °C for 48 hours, the glass coverslips containing PDMS micropillar arrays were peeled off from the negative molds and cut into small pieces. Fabricated PDMS micropillar array substrates were immersed in 100% ethanol and sonicated to restore collapsed micropillars, then dried with a critical point dryer (Samdri®-PVT-3D), then mounted on a 60 mm petri dish with a 15 mm hole in the center. Finally, PDMS micropillar arrays were functionalized using microcontact printing with fibronectin (50 µg/ml; Sigma-Aldrich) and Alexa-Fluor 647-conjugated fibrinogen (25 µg/ml; Life Technologies). Schematic illustration of micropillar fabrication and functionalization is presented in **Fig. S2**.

Microbubble attachment to VSMCs

Biotinylated VesselVue microbubbles (Sonovol) of diameter between 4-5 µm were used in this study. First, the microbubble solution was mixed with streptavidin (10 mg/ml, ThermoFisher) at a volume ratio of 20:1 for 30 min and kept at room temperature to form streptavidin-conjugated microbubbles via streptavidin-biotin binding. The streptavidin-conjugated microbubbles were washed twice with phosphate buffered saline (PBS, Gibco) to remove the unbound streptavidin. Next, biotinylated RGD peptides (2 mg/ml, Vividtide) were added to the streptavidin-coated

microbubble solution in the ratio of 2:21 for 20 min at room temperature, then 1.2 μL of the microbubble solution was collected from the top layer and mixed with 48.8 μL of cell culture medium. Cell culture medium was aspirated from the petri dish containing VSMCs on micropillars. The prepared microbubble solution was added on top of the micropillar arrays with adherent cells. The petri dish was then flipped for 10 min to allow microbubbles to bind to the adherent cells via floatation. Lastly, the petri dish was flipped back and gently washed to remove any unbound microbubbles. VSMCs with single microbubbles attached were selected for experimentation with ultrasound tweezers.

Image recognition-based quantification of partial actin deviation

The fluorescent images of F-actin were first skeletonized using a Canny edge detection (MATLAB 2020b). The actin CSK was detected by applying Hough transform (a line extraction technique for digital image processing (Ballard, 1981)) . Next, the Hough-Peaks associated with the Hough transform matrix were found. Finally, the lines associated with the Hough-Peaks were determined. The angle associated with the lines was used to calculate the orientation of the actin fiber associated with the detected line. The partial actin deviation with the detected lines were then calculated based on formula established in a previous study (Liu, Mollaeian, & Ren, 2018). Detailed pipeline is presented in **Fig. S8**.

2. **Supporting Figures.**

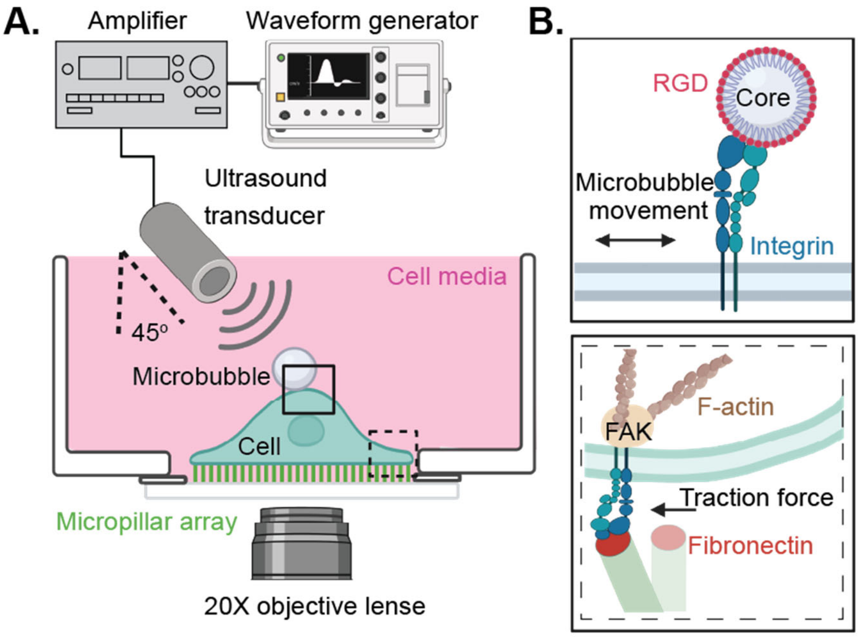

Figure S1. Schematic illustration of the ultrasound tweezers-based micromechanical system for single-cell mechanosensation study. A) Experimental apparatus including a function generator, a power amplifier, and an ultrasound transducer that applies ultrasound pulses to generate acoustic radiation force on the microbubble attaching on cell membrane causing its displacement. The cell is loaded on a PDMS micropillar array substrate and the cellular force response is observed with an inverted microscope. **B**) Schematic illustrations showing attachment of microbubble onto cell membrane via RGD-integrin binding (top panel), and cell traction force measurement based on the deflections of micropillars underneath the cell (bottom panel). Figure is created with BioRender.com.

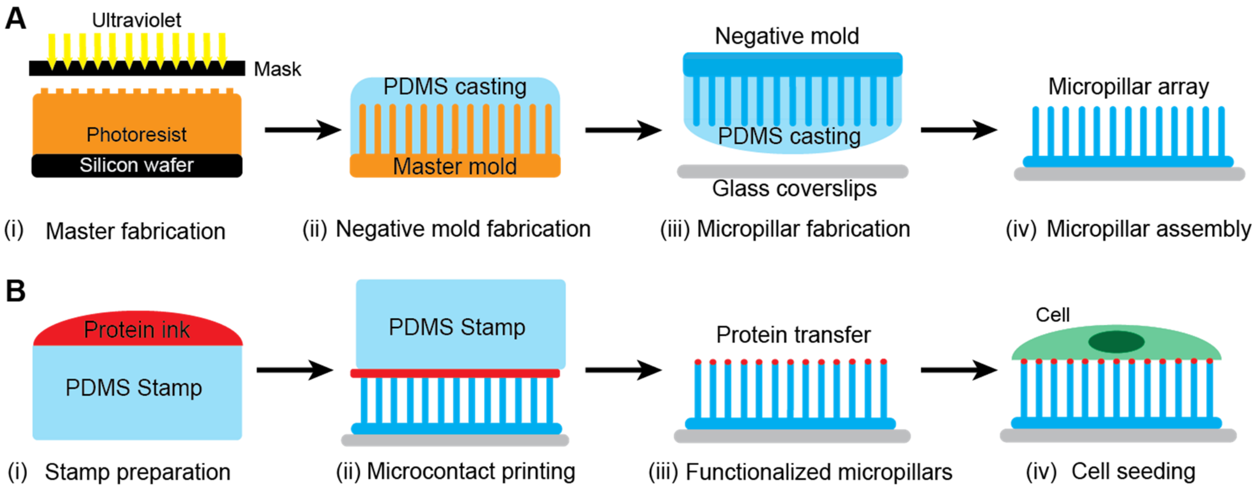

Figure S2: Micropillar array fabrication, microcontact printing, and cell seeding. **A**) Schematic illustration of the PDMS micropillar array fabrication procedure which includes (i) silicon master mold manufacturing using photolithography, (ii) creating PDMS negative mold, and (iii-iv) making PDMS micropillar array on a glass substrate from the negative molds though a soft lithography fabrication process. **B**) Schematic illustration of the cell seeding process, including (i) coating the PDMS stamp with protein solution containing fibronectin and fluorescence-conjugated fibrinogen adhesive proteins, (ii-iii) transferring the adhesive proteins onto the tips of the micropillars using a microcontact printing technique, and (iv) loading of cells onto the functionalized micropillar array substrate.

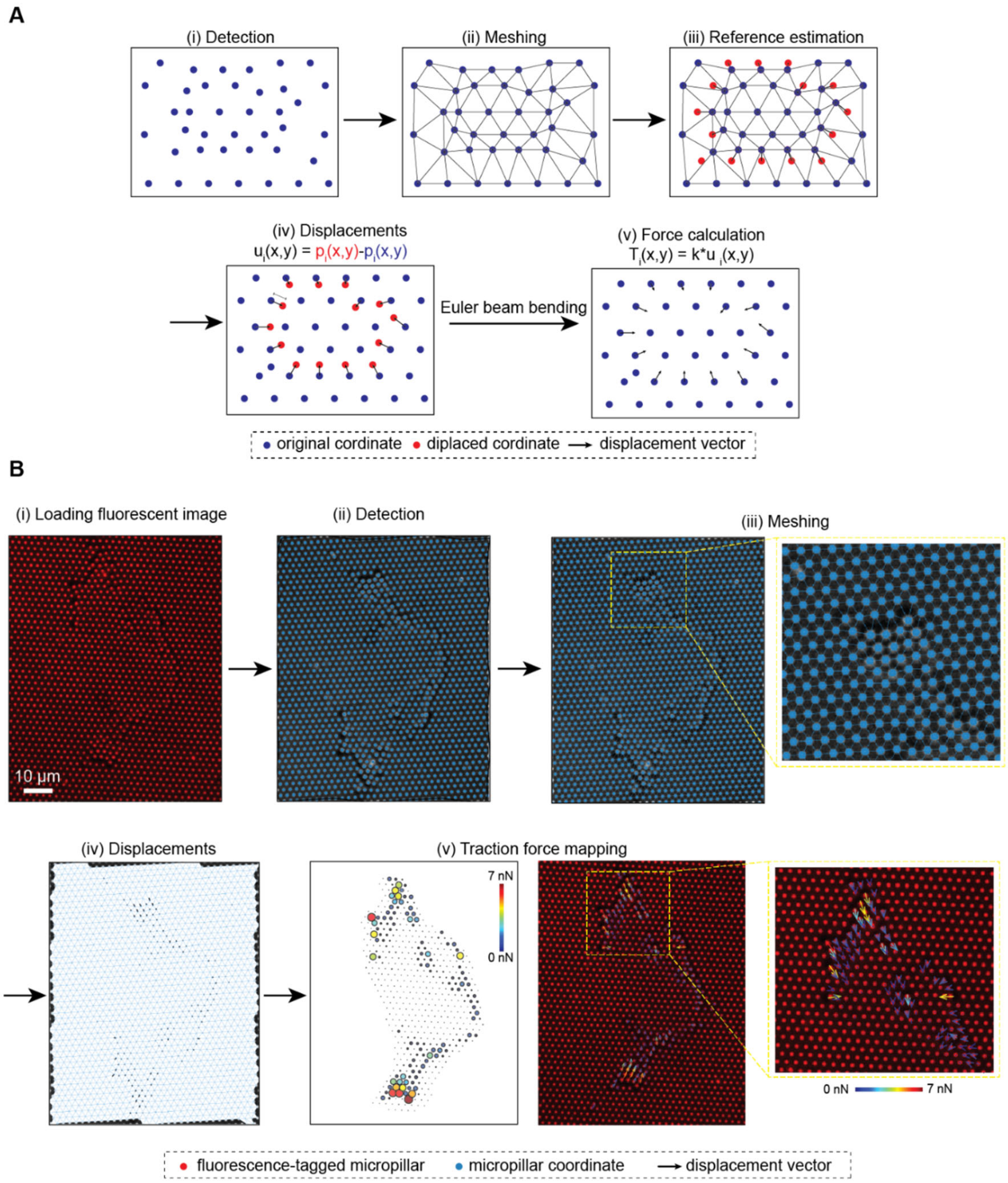

Figure S3: Traction force measurement using the PDMS micropillar array. A) The pipeline stages showing calculation of traction force using Cellogram software (open source). The software first detects the coordinate of single micropillar and presents them as a circle dot (i). Next, a mesh is generated to connect all the detected micropillars (ii). Energy minimization is performed to detect the reference position of the micropillars (iii). Displacement vector is obtained by subtracting the displaced coordinates with the reference coordinates. Finally, an elastic beam theory based on displacement and spring constant of the micropillars is applied to calculate the traction forces exerted by the cells. **B**) Example of the workflow showing traction force mapping from the fluorescent images (red channel) of the micropillars. The v panel shows a representative traction force heatmap (left) and a traction force vector map (right) of VSMC.

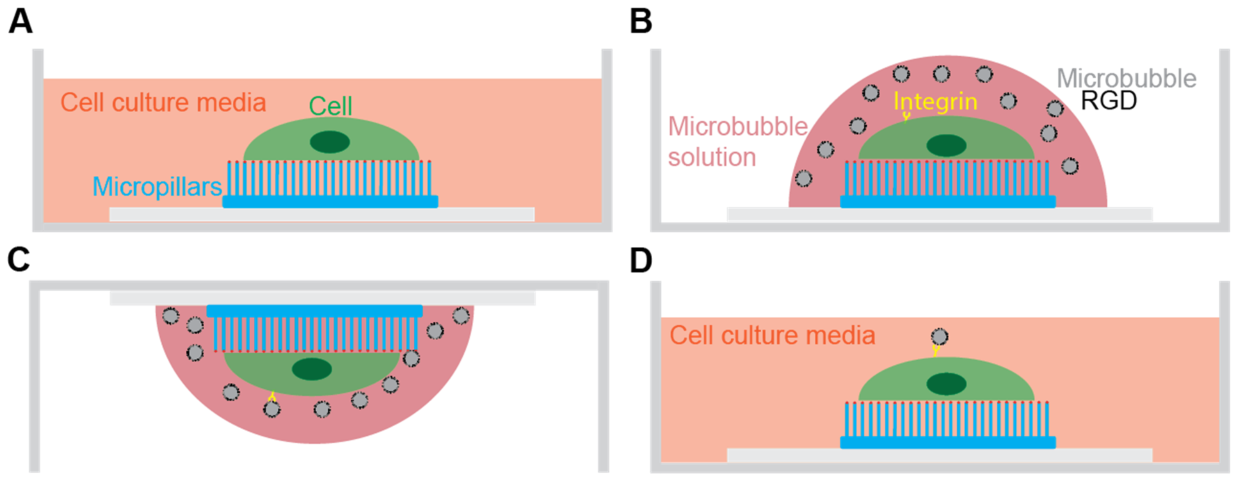

Figure S4: Microbubble attachment to cell. **A**) Single cells are seeded on a fibronectin-coated micropillar array substrate. **B**) Cell culture media is removed, and microbubble solution was added to the cells. **C**) The culture dish is flipped for 20 minutes to facilitate microbubble attachment via floatation. **D**) Unbounded bubbles are washed away with cell culture media twice, and cells bound with microbubbles are then suspended in fresh culture media for following ultrasound stimulation experimentation.

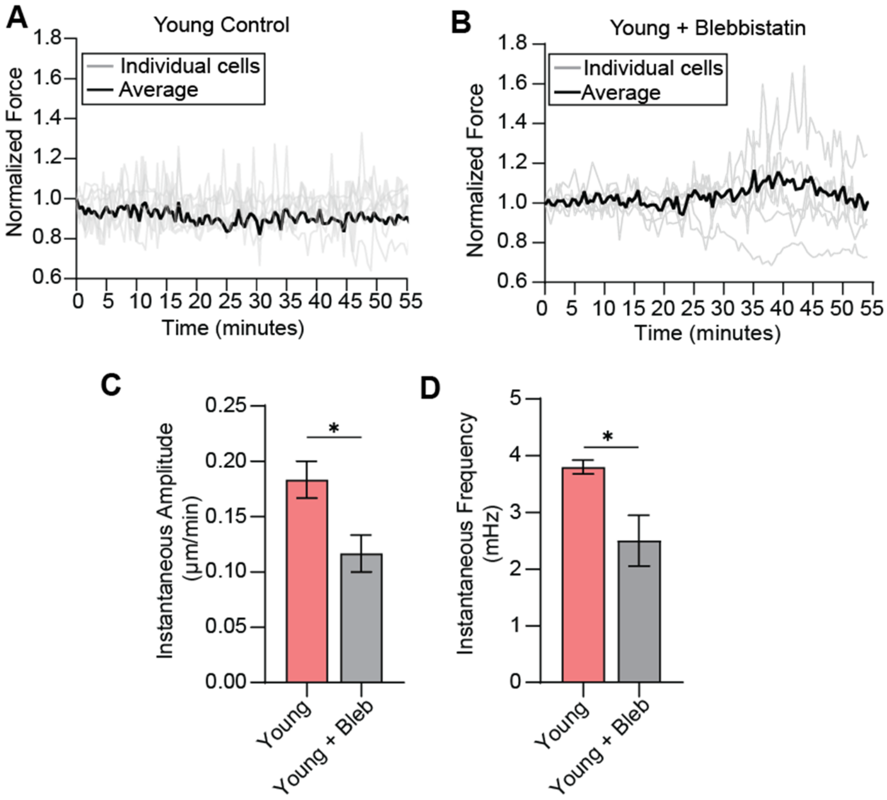

Figure S5: **Force dynamic response of VSMCs without ultrasound stimulation and treated with Blebbistatin with inhibited myosin-mediated contractility. A**) Temporal evolution of normalized traction force of VSMCs on PDMS micropillar array without ultrasound stimulation**.** Gray lines represent temporal force response of individual cells, and black line represents average force response of all measured cells (n=6). **B**) Dynamic force response of Blebbistatin treated young cells (n=5). **C**) Instantaneous amplitude and **D**) Instantaneous frequency of young versus young + Blebbistatin cells (n=5). In C, D, data are presented as mean values ± SEM, P-values were calculated using Student's t-test. * denotes p <0.05.

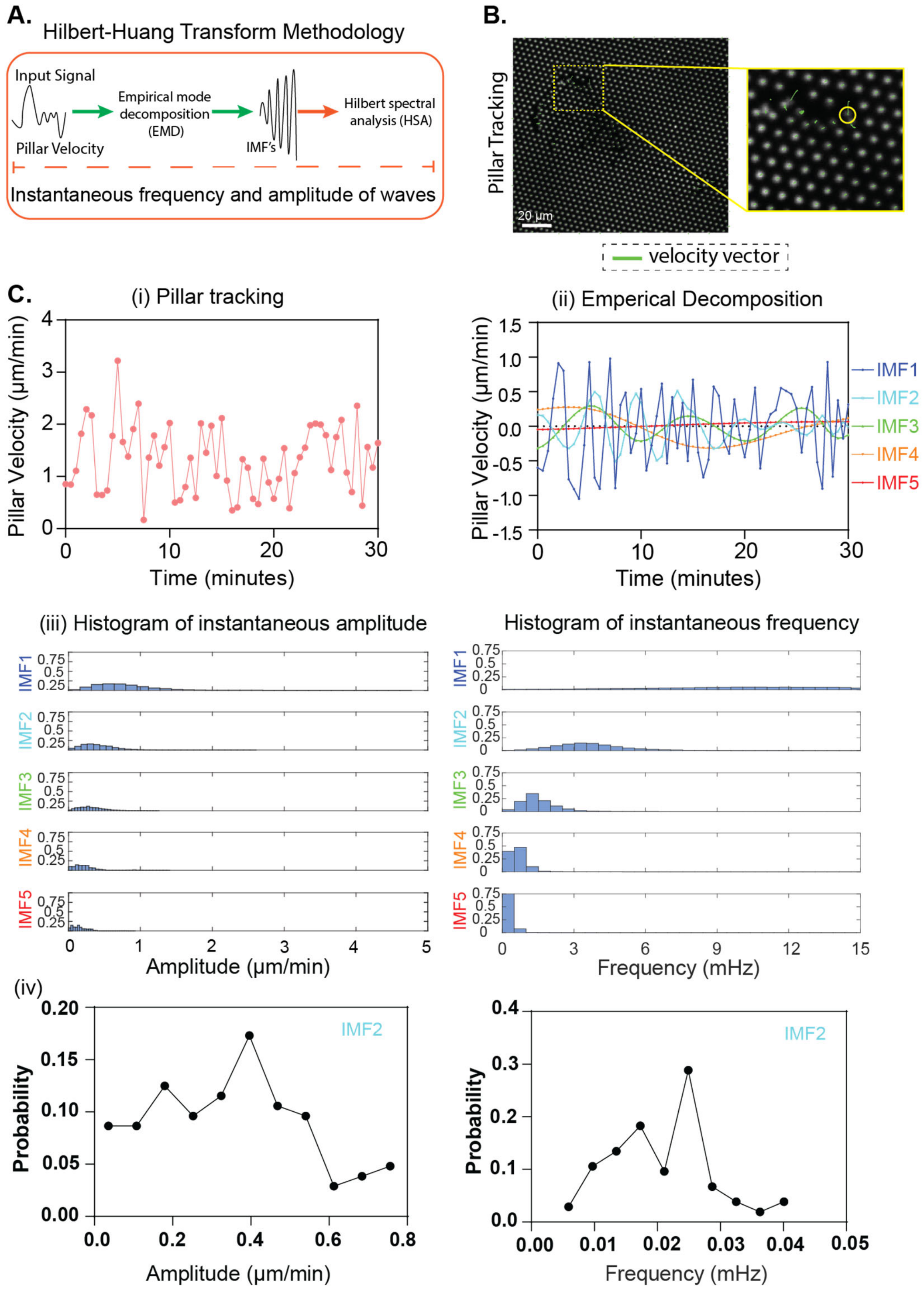

Figure S6: Instantaneous frequency spectrum analysis. A) Pipeline of Hilbert-Huang transform methodology, including tracking individual micropillar's velocity, performing empirical mode decomposition (EMD) of the velocity signal, and then applying Hilbert transform to the decomposed signal to get the instantaneous spectrum**. B)** Representative images showing displacement of individual pillars (white dots) and velocity (green arrows)**. C)** Application of EMD on the pillar velocity**. (i)** Pillar velocity variation over time serving as the input signal**. (ii)**

EMD separates the velocity time series into a finite and small numbers of component signals called intrinsic mode functions (IMFs). The number of IMFs was predetermined as 5 in this study based on the termination criterion, i.e. the variance of residual signal (equals to subtraction of the sum of IMF1 to IMF5 from the original signal) is less than 5% of that of the original signal. Hilbert Transform is applied to each IMF to compute instantaneous amplitude and instantaneous frequency of the micropillar's velocity signal. **(iii)** Histograms showing the instantaneous amplitude and instantaneous frequency in the signal from different IMFs. (iv) Examples of probability distributions of instantaneous properties (amplitude and frequency) derived from IMF2 of the micropillar's velocity signal.

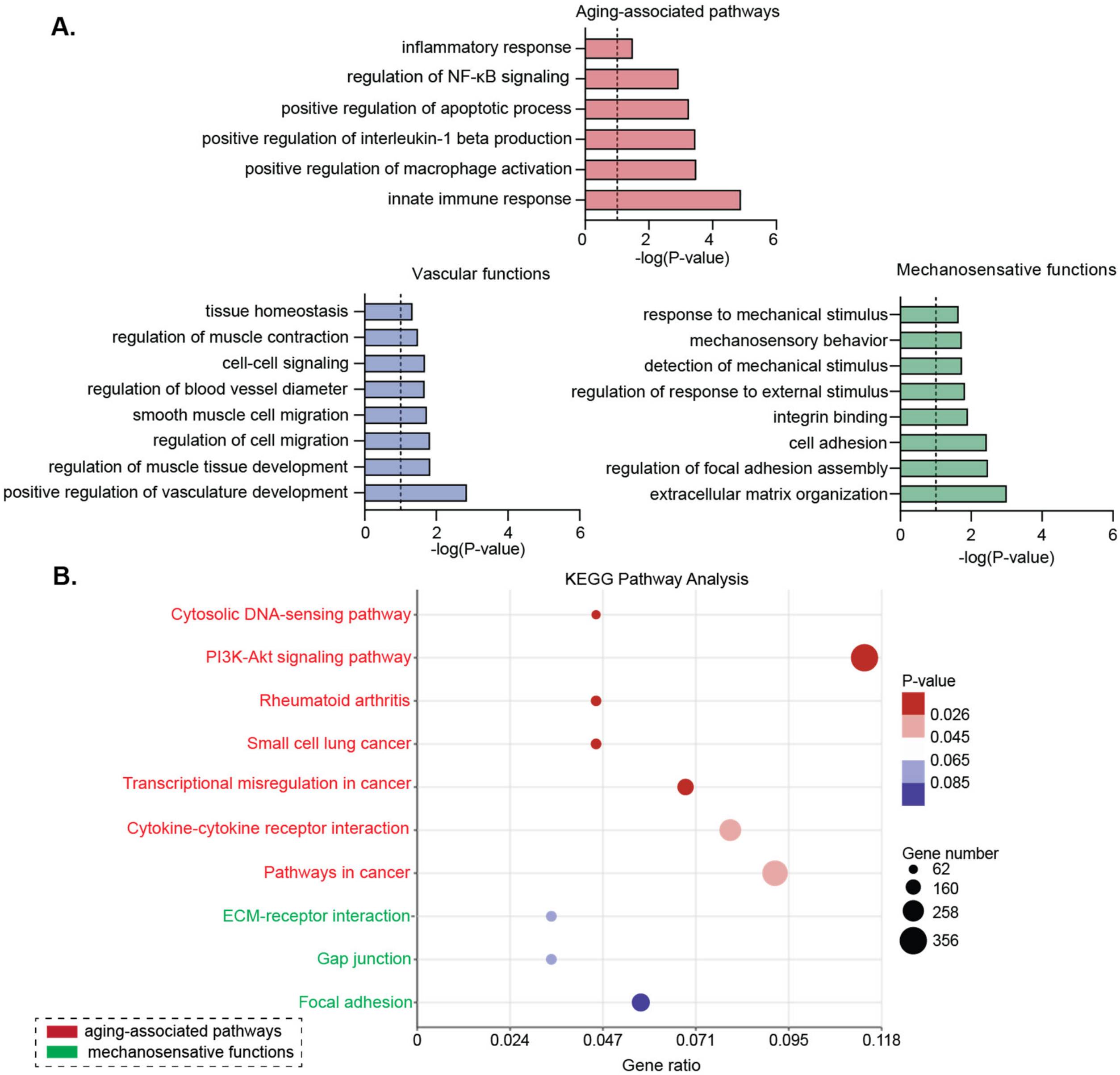

Figure S7. Pathway analysis of differentially expressed genes in young versus old VSMCs. A) GO enrichment analysis demonstrating alterations in VSMC biological processes with aging, based on the Gene Ontology Resource knowledgebase. The enriched pathways were categorized into aging-associated pathways, vascular functions, and mechanosensitive functions. **B)** KEGG pathway analysis performing on differentially expressed genes in young and old VSMCs (n=3).

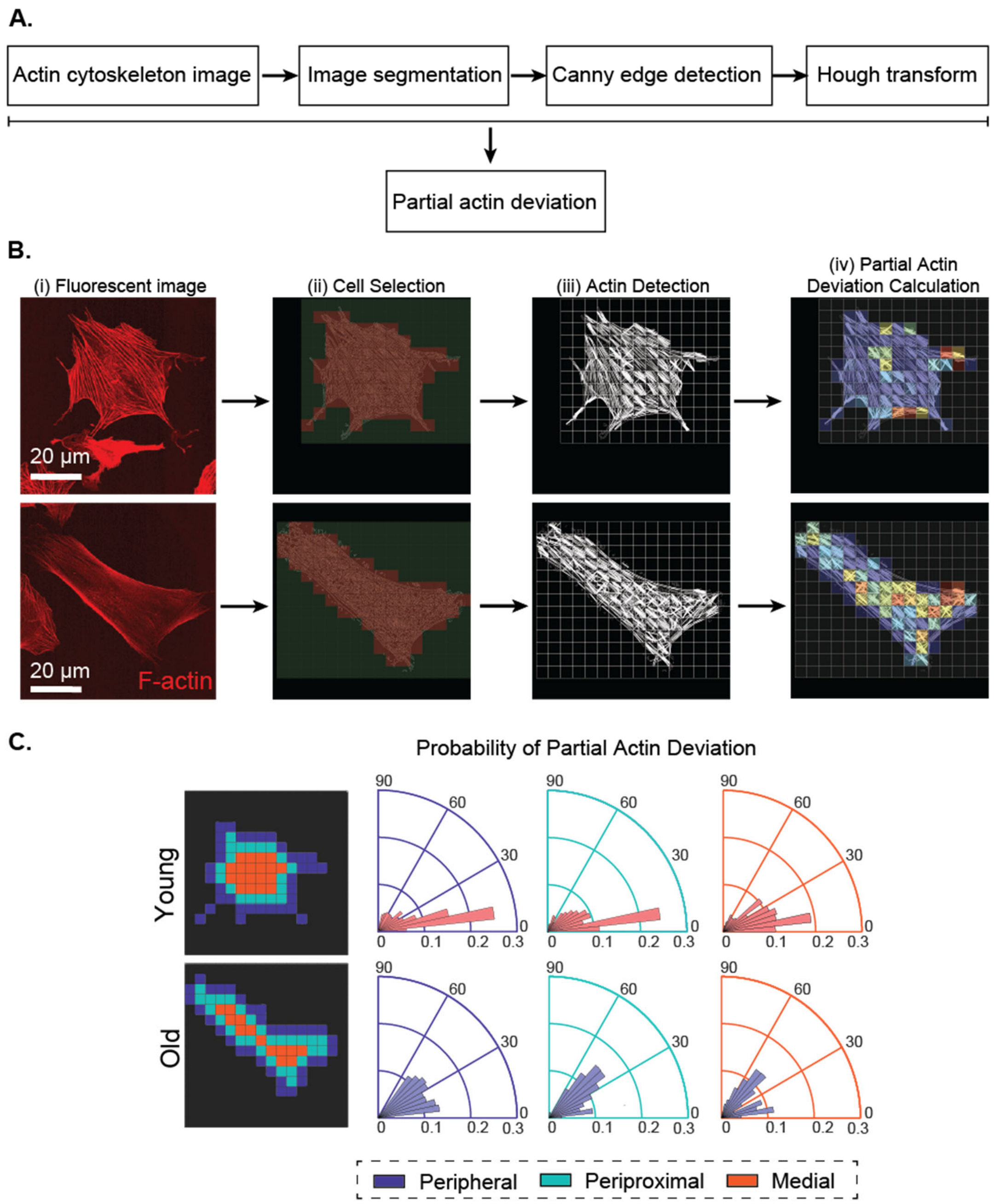

Figure S8. Subcellular F-actin analysis. A) Outline of image recognition-based actin CSK quantification method to calculate partial actin deviation. **B**) An example demonstrating subcellular actin analysis of young and old cells, including raw F-actin fluorescent image (i), cellular selection using boxes of 128x128 pixel (ii), F-actin fiber detection (iii), and partial actin deviation calculation. **C**) Polar histogram quantifying the subcellular distribution of the PADs in peripheral, periproximal and medial section of young and old cells as indicated.

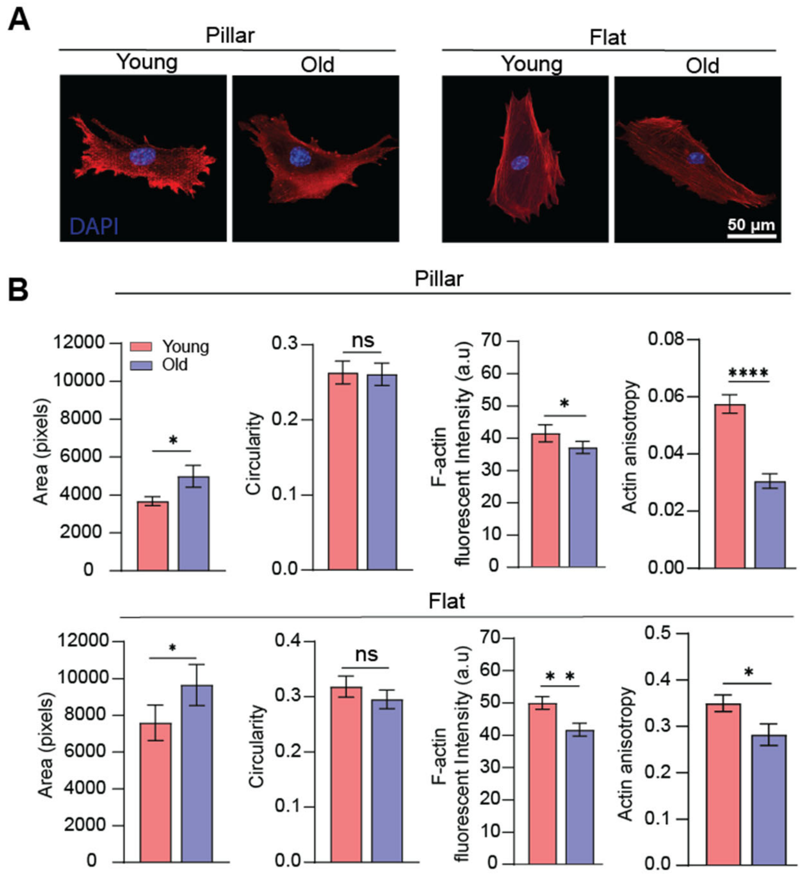

Figure S9. Comparison of VSMC morphology and actin arrangement on micropillar and flat substrates. **A**) Representative immunofluorescent staining images and **B**) quantification of cell morphology features (cell area and circularity), F-actin fluorescent intensity, and F-actin anisotropy of young and old VSMCs after 24-hour culture on micropillar and flat glass substrates (n=40). In B, data are presented as mean values ± SEM, P-values were calculated using Student's t-test. * denotes p <0.05, ** p<0.005, **** p<0.00005, and ns denotes no significant difference.

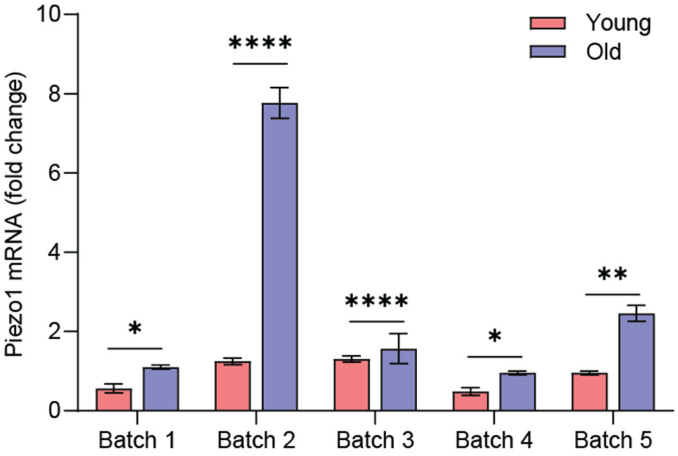

Figure S10. Compassion of Piezo1 gene expression in young and old VSCMs isolated from 5 different mice. qRT-PCR analyses were performed on four independent cultures (Batch 1-4) from the same vendor (Creative Bioarrays) and one culture (Batch 5) from a different vendor (Cell Biologics) to confirm Piezo1 expression in young and old VSMCs (n=3). Data are presented as mean values ± SEM, P-values were calculated using Student's t-test. * denotes $p<0.05$, ** $p<0.005$, **** $p<0.00005$.

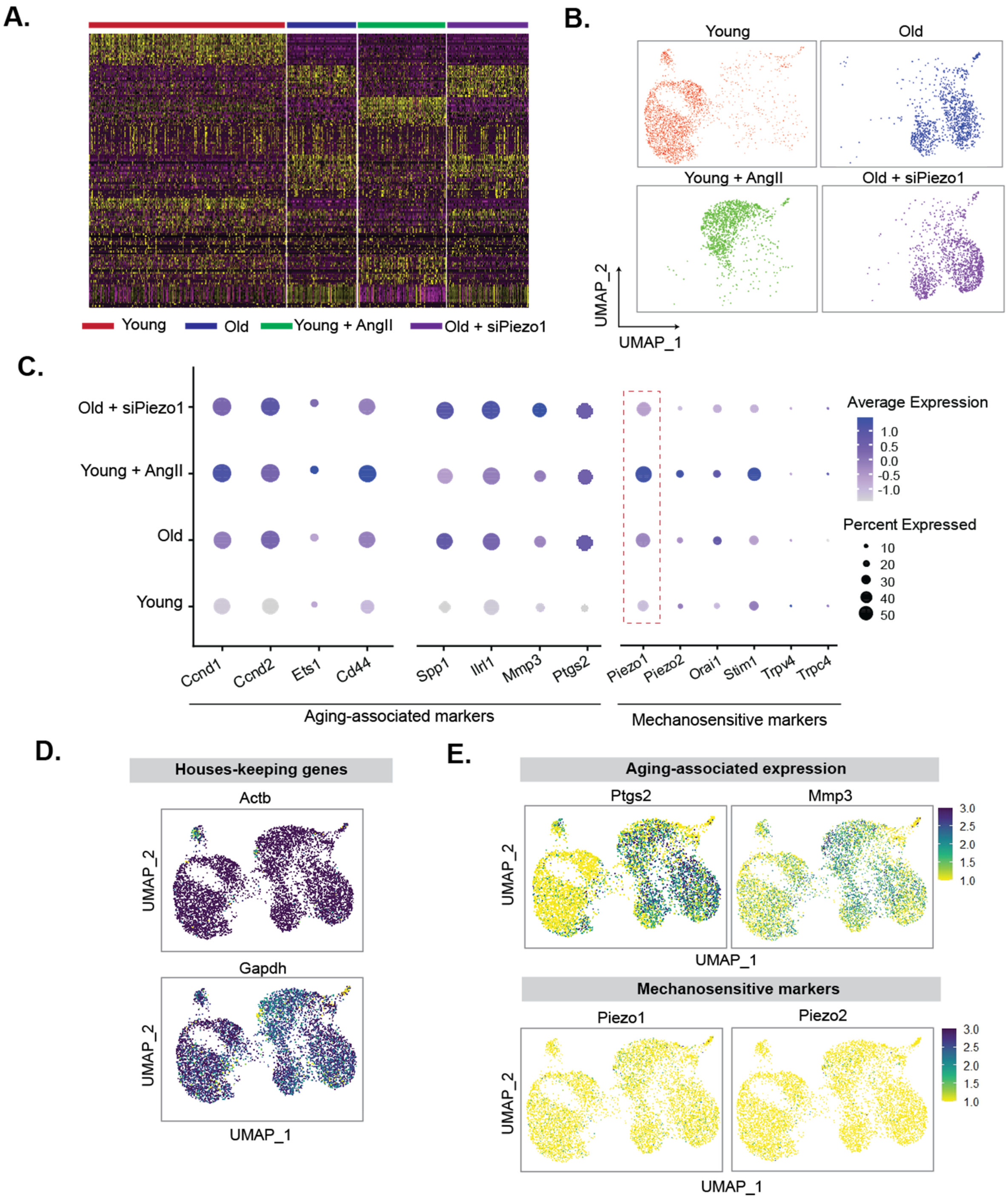

Figure S11. Single-cell RNA sequencing mapping of young, old, AngII-induced premature, and Piezo1-reversed aging VSMCs. A) Heatmap representation of top 20 differentially expressed genes across four cell clusters**. B)** UMAP projection of each VSMC subset labeled by obligo-tagged antibodies**. C)** Dot plot showing average and percent expression of genes encoded for aging-associated markers and mechanosensitive channels in the four cell clusters**. D)** Feature plots showing expression of housekeeping genes across the VSMC subsets. **E**) UMAP projection highlighting the expression of VSMC markers in cluster of young cells.

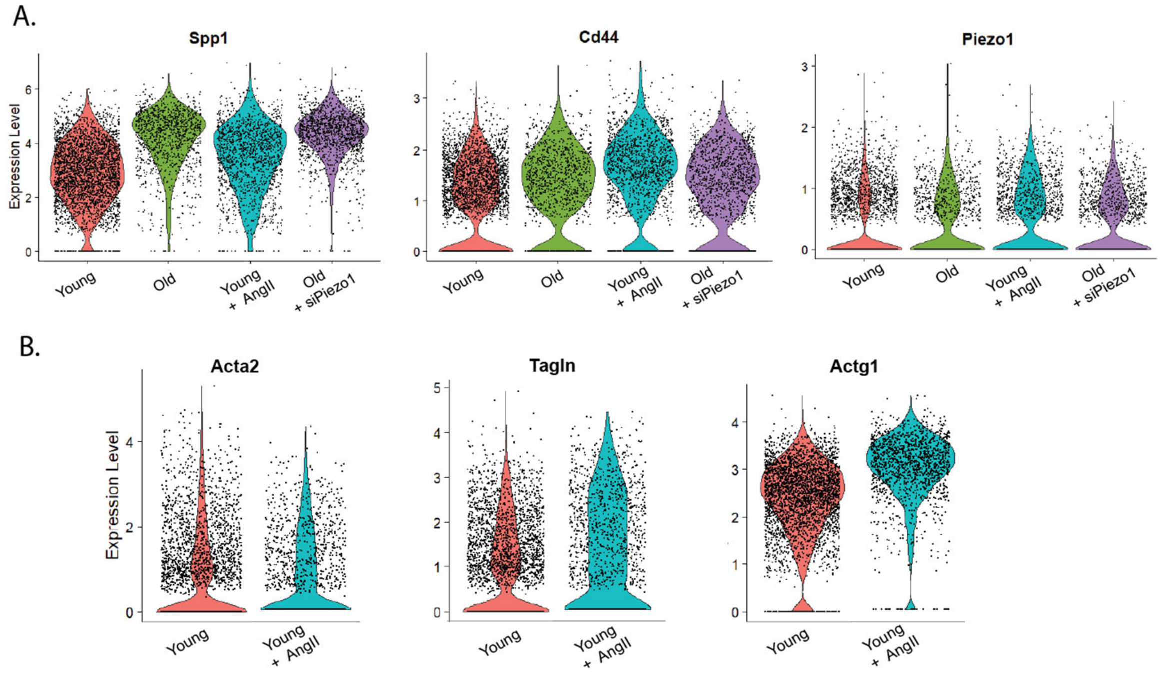

Figure S12. Expression of aging-associated markers and vascular cells markers in young, old, AngII-induced premature, and Piezo1-reversed aging VSMCs. A) Violin plots for markers of Spp1, Cd44, and Piezo1 among the four clusters. **B**) Violin plots quantifying expression of cytoskeletal genes in young versus young+AngII cluster.

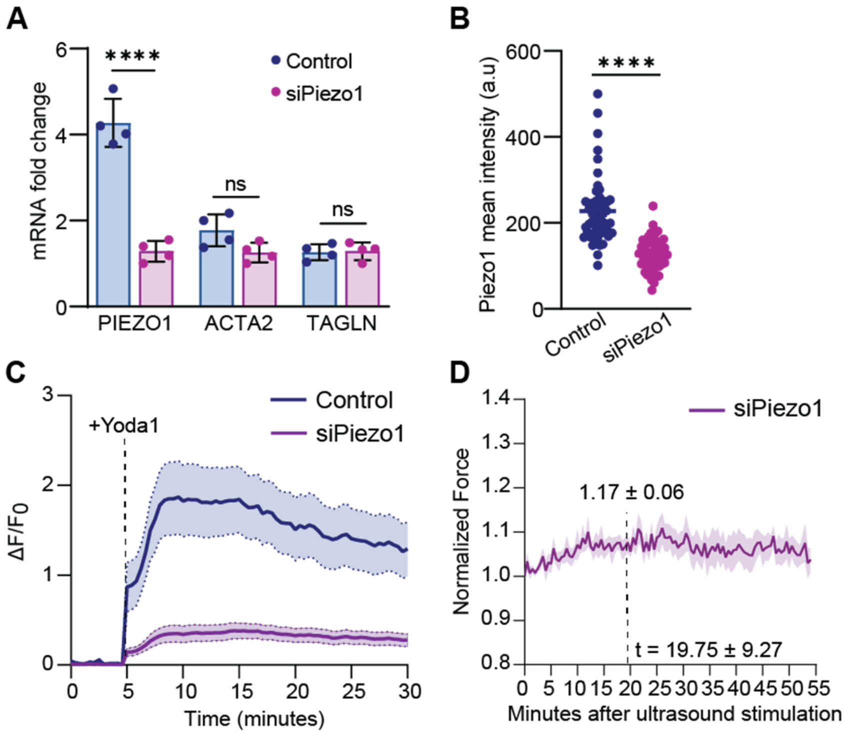

Figure S13. **Inhibition of Piezo1 in VSMCs with Silencer Pre-designed Piezo1 siRNA. A**) qRT-PCR analysis (n=4) of Piezo1, VSMC marker ACTA2 and TAGLN, and **B**) Immunofluorescent analysis (n=40 cells per group) of Piezo1 expression in old cells (control) and old cells transfected with Silencer Pre-designed Piezo1 siRNA (AM16708, Life Technologies). **C**) Time course measurement of Ca^{2+} response in VSMCs treated as indicated in response to Yoda1 stimulation (n=15). **D**) Normalized global traction force dynamics. Peak force response and response time (t) are indicated in the figure. (n=5). All statistical analysis was performed by Student's t-test and data are presented as mean values ± SEM. **** denotes p<0.00005.

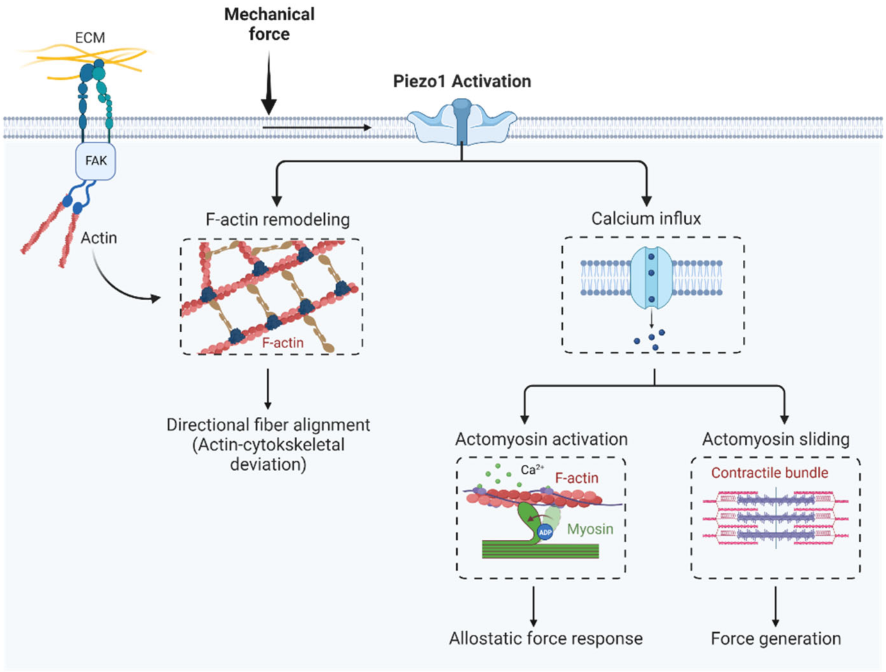

Figure S14. Schematic of Piezo1-dependent signaling pathway that mediates VSMC mechanosensation. The figure is created with BioRender.com.

3. Supporting Tables.

Table S1. Top 20 differentially expressed genes in each cell clusters.

	p_val	avg_log2FC	pct.1	pct.2	p_val_adj	cluster	gene
1	5.3E-307	0.6557306	0.992	0.892	1.267E-302	0	Ank3
2	8.1E-293	0.7170414	1	1	1.949E-288	0	Fth1
3	3.2E-280	0.9158702	0.985	0.906	7.749E-276	0	Fst
4	7.7E-238	1.0222305	0.925	0.771	1.854E-233	0	Bst2
5	3.3E-222	0.5593425	0.912	0.667	7.866E-218	0	Fgf10
6	6.7E-212	0.6905259	0.913	0.732	1.603E-207	0	Prkg1
7	5E-210	0.8503773	0.982	0.895	1.19E-205	0	Lcn2
8	3.8E-208	0.7900804	0.611	0.321	9.086E-204	0	Ly6a
9	2.2E-206	0.66095	0.989	0.915	5.232E-202	0	Slit2
10	1.3E-198	0.5062473	0.978	0.849	3.181E-194	0	Efna5
11	4.5E-196	0.5168283	0.999	0.965	1.08E-191	0	Pcdh9
12	2.5E-192	0.530423	1	1	6.009E-188	0	Spp1
13	5.8E-159	0.5068172	0.355	0.136	1.4E-154	0	Rspo2
14	6.6E-157	0.7826878	0.999	0.994	1.571E-152	0	Mgp
15	4.4E-144	0.5440384	0.948	0.85	1.06E-139	0	Mid1
16	1.6E-122	0.6350828	0.824	0.639	3.855E-118	0	Cemip
17	3.8E-111	0.5243386	0.603	0.392	9.101E-107	0	Dio2
18	2.8E-100	0.6105259	0.943	0.835	6.6389E-96	0	S100a4
19	1.28E-92	0.5879286	0.631	0.454	3.0552E-88	0	Gm2115
20	4.66E-57	0.5402494	0.754	0.626	1.1147E-52	0	Il1rl1
21	0	1.5027207	0.993	0.653	0	1	Hmgb2
22	0	1.3457143	0.922	0.384	0	1	Ube2c
23	0	1.283693	0.983	0.399	0	1	Pclaf
24	0	1.2613251	0.93	0.32	0	1	Top2a
25	0	1.195297	0.959	0.353	0	1	Birc5
26	0	1.1175162	0.954	0.385	0	1	Diaph3
27	0	1.0668737	0.941	0.425	0	1	Cdk1
28	0	1.0634269	0.992	0.724	0	1	Stmn1
29	0	0.9529572	0.876	0.282	0	1	Prc1
30	0	0.9491931	0.976	0.669	0	1	Cks2
31	0	0.9350377	1	0.987	0	1	H2afz
32	0	0.8767915	0.841	0.332	0	1	Cenpa
33	0	0.8576142	0.967	0.515	0	1	Smc2
34	0	0.8367646	0.923	0.35	0	1	Tpx2
35	0	0.8353595	0.82	0.29	0	1	Cenpf
36	0	0.7998212	0.852	0.207	0	1	Mki67
37	0	0.7813203	0.965	0.552	0	1	Tmpo

38	0	0.7760849	0.66	0.201	0	1	Hist1h1b
39	0	0.766269	0.996	0.891	0	1	Cks1b
40	2.6E-234	0.8115534	0.992	0.908	6.255E-230	1	Fst
41	3.6E-300	0.8027645	0.939	0.725	8.555E-296	2	Pmepa1
42	1.8E-264	0.8753445	0.944	0.798	4.268E-260	2	Ccbe1
43	3E-163	0.7263417	0.982	0.953	7.113E-159	2	Ccl7
44	1.3E-161	0.6652392	0.804	0.641	3.139E-157	2	Dpysl3
45	1E-151	0.7878299	0.898	0.801	2.425E-147	2	Col3a1
46	9.9E-146	0.6361331	0.791	0.621	2.358E-141	2	Thbs2
47	1.8E-141	0.7815721	0.969	0.919	4.391E-137	2	Tnc
48	2.6E-135	0.7221043	0.949	0.893	6.225E-131	2	Col5a2
49	2.7E-132	0.6656529	0.897	0.805	6.362E-128	2	Adm
50	3.1E-129	0.665435	0.697	0.535	7.314E-125	2	Cxcl12
51	3.7E-129	0.7324036	0.603	0.332	8.804E-125	2	Xist
52	2.9E-121	0.6561888	0.425	0.215	7.056E-117	2	Nlgn1
53	3.7E-117	0.579762	0.745	0.557	8.89E-113	2	Nupr1
54	2.1E-114	0.5833226	0.772	0.593	5.085E-110	2	Adam12
55	7.6E-111	0.7355549	0.88	0.795	1.825E-106	2	Dcn
56	2.6E-102	0.6268213	0.659	0.494	6.2573E-98	2	8030451A03Ri k
57	2.11E-94	0.5754378	0.72	0.606	5.0625E-90	2	Vegfc
58	2.48E-71	0.6133284	0.785	0.707	5.9448E-67	2	Col12a1
59	5.22E-55	0.6237045	0.833	0.748	1.2506E-50	2	Mmp3
60	1.25E-32	0.6696109	0.514	0.422	2.9835E-28	2	Mmp10
61	0	1.7073489	0.992	0.404	0	3	Pclaf
62	0	1.5167451	0.994	0.656	0	3	Hmgb2
63	0	1.4390882	0.921	0.328	0	3	Top2a
64	0	1.3156718	0.913	0.392	0	3	Ube2c
65	0	1.2337388	0.943	0.363	0	3	Birc5
66	0	1.178423	0.943	0.431	0	3	Cdk1
67	0	1.1754854	0.994	0.727	0	3	Stmn1
68	0	1.1573053	0.877	0.289	0	3	Prc1
69	0	1.1178779	0.978	0.673	0	3	Cks2
70	0	1.0856456	0.966	0.521	0	3	Smc2
71	0	1.045455	1	0.987	0	3	H2afz
72	0	1.0352973	0.928	0.396	0	3	Diaph3
73	0	1.007972	0.646	0.209	0	3	Hist1h1b
74	0	0.999331	0.899	0.36	0	3	Tpx2
75	0	0.9985596	0.785	0.302	0	3	Cenpf
76	0	0.9881123	0.829	0.218	0	3	Mki67
77	0	0.9731648	0.874	0.28	0	3	Rrm2
78	0	0.9527849	0.997	0.892	0	3	Cks1b

79	0	0.95054	0.97	0.652	0	3	Dek
80	1.7E-224	1.2009684	0.654	0.327	4.098E-220	3	Xist
81	0	1.9154142	0.925	0.485	0	4	Clu
82	0	1.5039675	0.989	0.899	0	4	Lcn2
83	0	1.345258	0.774	0.378	0	4	Hdac9
84	0	1.343006	0.998	0.898	0	4	Cp
85	0	1.3156262	0.919	0.455	0	4	Sned1
86	0	1.2900756	1	0.967	0	4	Pcdh9
87	0	1.2395748	0.964	0.622	0	4	Serpina3g
88	0	1.2354389	0.997	0.885	0	4	Trps1
89	0	1.2068607	0.994	0.855	0	4	Fndc3a
90	0	1.1885939	0.993	0.901	0	4	Alcam
91	0	1.1879311	0.993	0.913	0	4	Rasa1
92	0	1.1476295	0.994	0.874	0	4	Dock4
93	0	1.1425267	0.999	0.897	0	4	Ank3
94	0	1.1245001	0.989	0.829	0	4	Fmnl2
95	0	1.1224049	0.919	0.51	0	4	C3
96	9.7E-279	1.1456705	0.81	0.454	2.319E-274	4	Serpina3n
97	2.8E-191	1.2198538	0.261	0.059	6.699E-187	4	Csmd1
98	2.7E-164	1.6647213	0.333	0.107	6.452E-160	4	Nxph1
99	8.4E-138	1.1681887	0.929	0.826	2.008E-133	4	Saa3
100	4.08E-46	1.1272401	0.46	0.309	9.7584E-42	4	Ptn
101	0	2.3223621	0.963	0.637	0	5	Cryab
102	0	1.9629057	0.759	0.282	0	5	Serpinb2
103	0	1.4597856	0.771	0.144	0	5	Gpr39
104	0	1.3617318	0.987	0.905	0	5	Col1a1
105	0	1.2943854	0.985	0.867	0	5	Cdh2
106	0	1.283379	0.994	0.893	0	5	Fbln2
107	0	1.2688528	0.998	0.981	0	5	Sparc
108	0	1.1826005	0.549	0.122	0	5	Olr1
109	2.4E-305	1.4954207	0.998	0.991	5.694E-301	5	Timp1
110	1.8E-301	1.4419243	0.924	0.671	4.2E-297	5	Lox
111	2.8E-281	1.4272059	0.898	0.657	6.692E-277	5	Rbp1
112	6E-264	1.2579241	0.94	0.7	1.431E-259	5	Pdzrn3
113	7E-234	1.1941052	0.902	0.67	1.68E-229	5	Ncam1
114	5.4E-193	1.071531	0.854	0.553	1.296E-188	5	Serpine1
115	4E-151	1.2366378	0.889	0.693	9.641E-147	5	Tagln
116	4.9E-137	2.3419529	0.657	0.398	1.181E-132	5	Ctla2a
117	2E-129	1.0813566	0.927	0.804	4.778E-125	5	Col3a1
118	8E-116	1.1513701	0.816	0.6	1.911E-111	5	Acta2
119	6.42E-75	1.1246477	0.624	0.455	1.537E-70	5	Timp3

120	5.31E-71	1.0574175	0.901	0.85	1.2717E-66	5	Slit3
121	3.5E-307	1.4402818	0.995	0.885	8.29E-303	6	Grem1
122	1.1E-300	1.1897933	0.965	0.724	2.747E-296	6	Vegfa
123	3.5E-300	2.474319	0.616	0.18	8.479E-296	6	Il11
124	1.6E-291	1.4657168	0.998	0.962	3.94E-287	6	Odc1
125	8E-290	1.2670822	0.911	0.551	1.905E-285	6	Arap2
126	4.1E-275	1.1414834	0.979	0.854	9.819E-271	6	Ptges
127	3.5E-271	1.3402844	0.961	0.77	8.393E-267	6	Mmd
128	4.5E-269	1.2131584	0.993	0.909	1.071E-264	6	Vcan
129	1E-213	1.3073207	0.986	0.898	2.504E-209	6	Ptgs2
130	5.8E-206	1.5105777	0.644	0.249	1.378E-201	6	Fmo2
131	4.9E-204	1.4312783	0.956	0.772	1.175E-199	6	Sema3a
132	4.2E-190	1.1457064	0.995	0.954	9.984E-186	6	Ccl7
133	3E-189	1.1319275	0.989	0.947	7.157E-185	6	Ier3
134	4.3E-176	1.1254968	0.823	0.566	1.034E-171	6	Lrrk2
135	1.2E-152	1.7448412	0.263	0.052	2.848E-148	6	Igfbp5
136	1.2E-99	1.3540361	0.628	0.393	2.9722E-95	6	Gzme
137	5.53E-97	1.5559084	0.573	0.308	1.3245E-92	6	S100a8
138	8.34E-75	1.3944589	0.648	0.425	1.9961E-70	6	Cxcl1
139	5.48E-70	1.124632	0.71	0.529	1.3128E-65	6	Nrg1
140	2.31E-59	1.1317996	0.502	0.309	5.5367E-55	6	Penk
141	1.6E-305	1.0918669	1	0.992	3.95E-301	7	Cald1
142	1.1E-277	1.4383815	0.722	0.231	2.531E-273	7	Sema3e
143	4.5E-272	1.1936854	0.633	0.179	1.083E-267	7	Mylk
144	2.3E-259	1.0310689	0.687	0.208	5.534E-255	7	Klk8
145	2.2E-258	1.2303757	1	1	5.349E-254	7	Tmsb4x
146	5.2E-250	1.4095224	0.945	0.558	1.255E-245	7	Serpine1
147	1.4E-248	1.0968671	1	0.995	3.283E-244	7	Tpm1
148	2.4E-232	1.4754727	0.961	0.666	5.842E-228	7	Akap12
149	3.4E-214	1.2719305	0.88	0.436	8.035E-210	7	Ccn2
150	4.3E-211	1.4377245	0.913	0.568	1.034E-206	7	Sorbs2
151	8.3E-206	1.9490347	0.946	0.696	1.998E-201	7	Tagln
152	1.3E-193	1.2681731	0.776	0.365	3.217E-189	7	Myl9
153	5.5E-184	1.0884038	0.983	0.91	1.322E-179	7	Cited2
154	2.3E-171	1.1459917	0.998	0.988	5.397E-167	7	Dstn
155	4.7E-169	1.0388402	0.998	0.98	1.131E-164	7	Thbs1
156	8.5E-169	1.9077996	0.897	0.602	2.032E-164	7	Acta2
157	2.4E-161	1.0880323	0.924	0.696	5.631E-157	7	Peg3
158	4.2E-144	1.2713284	0.795	0.413	1.017E-139	7	Mmp10
159	2.3E-99	1.2457408	0.549	0.251	5.5118E-95	7	Ankrd1
160	1.69E-55	1.1101029	0.539	0.313	4.0377E-51	7	S100a8

161	1.7E-162	0.6657443	0.994	0.998	3.992E-158	8	Rpl37a
162	2.8E-150	0.7093597	1	1	6.747E-146	8	Rps27a
163	8E-150	0.6987494	1	0.999	1.925E-145	8	Rpl30
164	2E-146	0.8010279	0.997	0.998	4.748E-142	8	Rpl37
165	8.3E-137	0.7414211	0.997	0.999	1.988E-132	8	Ftl1
166	8.9E-136	0.7050528	0.976	0.992	2.122E-131	8	Elob
167	2.3E-133	0.7610817	1	1	5.406E-129	8	S100a6
168	4.9E-132	0.8395673	1	0.998	1.162E-127	8	Tmsb10
169	7.7E-132	0.6725072	0.974	0.992	1.841E-127	8	Atp5l
170	6.6E-131	0.7000271	0.992	0.999	1.591E-126	8	Rps21
171	1.7E-121	0.7222746	0.988	0.996	4.163E-117	8	Serf2
172	1.1E-104	0.6742795	0.98	0.993	2.567E-100	8	Ndufa4
173	3.5E-101	0.6787356	0.967	0.992	8.3093E-97	8	Gng5
174	4.04E-87	0.729732	0.953	0.985	9.6645E-83	8	Tbca
175	5.18E-84	0.7601877	0.979	0.987	1.2407E-79	8	S100a10
176	3.65E-74	0.6665599	0.997	0.999	8.7379E-70	8	S100a11
177	7.46E-70	0.8667902	0.943	0.958	1.7853E-65	8	2410006H16Rik
178	5.43E-63	0.6902259	0.986	0.992	1.3003E-58	8	Timp1
179	7.08E-29	1.1158309	0.392	0.233	1.6949E-24	8	Crct1
180	2.11E-28	0.8246574	0.753	0.661	5.0628E-24	8	Cryab
181	2.5E-276	1.1932944	0.833	0.179	6.009E-272	9	Gpr39
182	1E-157	1.9566023	0.979	0.655	2.478E-153	9	Cryab
183	4.2E-153	1.4328909	0.837	0.308	1.01E-148	9	Serpinb2
184	3.9E-151	1.0084478	0.913	0.349	9.325E-147	9	Prc1
185	2.1E-148	1.3097453	0.988	0.681	4.944E-144	9	Ncam1
186	5.9E-147	1.2171918	0.991	0.466	1.424E-142	9	Pclaf
187	1.9E-138	1.4887551	0.969	0.684	4.668E-134	9	Lox
188	4.7E-138	1.1137613	0.995	0.873	1.13E-133	9	Cdh2
189	1.5E-136	1.1509646	0.917	0.39	3.605E-132	9	Top2a
190	1.1E-124	1.1343599	0.934	0.446	2.544E-120	9	Ube2c
191	2E-122	1.0270089	0.998	0.899	4.871E-118	9	Fbln2
192	9.4E-122	1.1595848	0.972	0.669	2.252E-117	9	Rbp1
193	2.4E-121	1.1765776	0.998	0.91	5.789E-117	9	Col1a1
194	2.3E-111	1.1885268	1	0.991	5.606E-107	9	Timp1
195	1.8E-109	1.050082	0.818	0.399	4.407E-105	9	Ebf1
196	9.3E-109	1.0208183	0.991	0.692	2.234E-104	9	Hmgb2
197	1.53E-98	1.0246844	0.972	0.714	3.6632E-94	9	Pdzrn3
198	4.03E-79	1.0359243	0.642	0.257	9.6543E-75	9	Ankrd1
199	1.32E-68	1.8324663	0.733	0.411	3.1534E-64	9	Ctla2a
200	1.05E-44	1.4139047	0.693	0.406	2.5214E-40	9	Cxcl5
201	7.43E-71	2.0073567	1	0.999	1.7781E-66	10	mt-Co3

202	1.11E-70	1.9403774	1	0.999	2.6535E-66	10	mt-Co2
203	2.24E-70	1.7383498	1	0.997	5.3628E-66	10	mt-Nd1
204	4.46E-70	2.066748	1	0.995	1.0685E-65	10	mt-Nd2
205	1.46E-69	2.2019315	1	0.999	3.4908E-65	10	mt-Atp6
206	4.55E-69	1.8049163	1	0.998	1.0903E-64	10	mt-Cytb
207	7.08E-69	1.8667862	1	0.997	1.695E-64	10	mt-Nd4
208	1.72E-66	1.7334887	1	0.999	4.1159E-62	10	mt-Co1
209	3.58E-58	1.881212	0.991	0.934	8.5666E-54	10	mt-Nd5
210	6.44E-56	2.3777174	1	0.997	1.5408E-51	10	Malat1
211	6.13E-54	1.8370802	0.974	0.868	1.4674E-49	10	mt-Nd4l
212	3.86E-53	1.9800182	0.991	0.964	9.2479E-49	10	Gm42418
213	5.91E-48	1.6745682	0.991	0.97	1.4143E-43	10	Zfpm2
214	3.75E-46	1.7439702	0.965	0.89	8.9875E-42	10	Neat1
215	2.38E-35	1.6733399	0.86	0.661	5.6879E-31	10	mt-Nd6
216	3.91E-33	1.698081	0.754	0.459	9.355E-29	10	Fam227b
217	2.62E-22	2.1779508	0.728	0.516	6.2705E-18	10	8030451A03Rik
218	5.15E-18	1.6794236	0.807	0.703	1.232E-13	10	Lingo2
219	4.46E-17	1.7394994	0.789	0.722	1.0688E-12	10	Kcnq5
220	9.77E-08	1.6842032	0.614	0.539	0.002339	10	Gm8113
221	1.95E-61	3.8286018	1	0.999	4.676E-57	11	mt-Co1
222	2.05E-61	4.0784845	1	0.995	4.9004E-57	11	mt-Nd2
223	2.06E-61	3.8514505	1	0.997	4.9207E-57	11	mt-Nd4
224	2.24E-61	4.0726785	1	0.999	5.3557E-57	11	mt-Co3
225	3.15E-61	3.9810179	1	0.999	7.5417E-57	11	mt-Co2
226	8.58E-61	3.7024065	1	0.998	2.0546E-56	11	mt-Cytb
227	1E-60	3.9541511	1	0.934	2.4041E-56	11	mt-Nd5
228	1.85E-59	3.7507625	1	0.964	4.4401E-55	11	Gm42418
229	4.01E-59	3.8763501	1	0.997	9.6011E-55	11	mt-Nd1
230	1.8E-58	4.259589	1	0.999	4.3067E-54	11	mt-Atp6
231	8.53E-48	3.5856029	0.935	0.869	2.0414E-43	11	mt-Nd4l
232	2.88E-47	3.8792595	0.935	0.944	6.8855E-43	11	mt-Nd3
233	4.46E-32	2.9235095	0.685	0.312	1.0679E-27	11	mt-Atp8
234	1.95E-21	3.2847305	0.739	0.662	4.6692E-17	11	mt-Nd6
235	1.64E-13	0.4209353	0.141	0.674	3.9339E-09	11	Mrpl15
236	1.2E-11	2.1056757	0.533	0.37	2.8843E-07	11	AY036118
237	6.3E-07	0.5254814	0.217	0.65	0.01507902	11	Gphn
238	1.2E-05	1.9379195	0.413	0.326	0.2865198	11	Camk1d
239	2.56E-05	1.917836	0.478	0.465	0.6121998	11	Cmss1
240	0.002215	0.4934915	0.109	0.287	1	11	Shb

Table S2. List of primary and secondary antibodies.

**Antibody name**	**Catalog number**	**Vendor**	**Dilution ratio**
Anti-rabbit IgG (H+L), F(ab')2 Fragment (Alexa Fluor® 647 Conjugate)	4414S	Cell Signaling Technology	1:500-1:2000
Anti-Mouse IgG (H+L), CF™ 555 antibody produced in goat,~2 mg/mL, affinity isolated antibody	SAB4600066	Sigma-Aldrich	1:1000-1:2000
DAPI			1:500-1:1000
gamma Actin	PA1-16890	Invitrogen	1:10-1:500
MYH11	PA5-116524	Invitrogen	1:50-1:200
Phalloidin	A12379	Invitrogen	1:50
α-Smooth Muscle Actin	48938S	Cell Signaling Technology	1:200
Piezo1	NBP178446	Novus Biologicals	1:25

Table S3. Primer sequences used in qRT-PCR.

Gene	Forward	Reverse
ACTB	CATTGCTGACAGGATGCAGAAGG	TGCTGGAAGGTGGACAGTGAGG
ACTA2	TGCTGACAGAGGCACCACTGAA	CAGTTGTACGTCCAGAGGCATAG
ACTG2	CATTGCTGACAGGATGCAGAAGG	AGCCGCCAATCCAGACTGAGTA
ACTN2	ATCGCCATCATCTGGTTCCC	GCCTTGAACTGCTCATGTGCAG
MYH11	GCAACTACAGGCTGAGAGGAAG	TCAGCCGTGACCTTCTCTAGCT
RAC1	GGACACCATTGAGAAGCTGAAGG	GTCTTGAGTCCTCGCTGTGTGA
TAGLN	GCAGATGGAACAGGTGGCTCAA	CCCAAAGCCATTAGAGTCCTCTG
p21	TCGCTGTCTTGCACTCTGGTGT	CCAATCTGCGCTTGGAGTGATAG
p16	TGTTGAGGCTAGAGAGGATCTTG	CGAATCTGCACCGTAGTTGAGC
p53	CATGTACCTCCAGCAAGCCACA	AACGGTAAGGGTGATGGCGATG
COL1A	CCTCAGGGTATTGCTGGACAAC	CAGAAGGACCTTGTTTGCCAGG
COL3A	GACCAAAAGGTGATGCTGGACAG	CAAGACCTCGTGCTCCAGTTAG
COL6A	GACACCTCTCAGTGTGCTCTGT	GCGATAAGCCTTGGCAGGAAATG
TNFA	GGTGCCTATGTCTCAGCCTCTT	GCCATAGAACTGATGAGAGGGAG
IL1B	TGGACCTTCCAGGATGAGGACA	GTTCATCTCGGAGCCTGTAGTG
PIEZO1	ATCGCCATCATCTGGTTCCC	CTAGCTTGAGGGTGACGGTG
PIEZO2	GCACTCTACCTCAGGAAGACTG	CAAAGCTGTGCCACCAGGTTCT
ORAI1	GTTACTCCGAGGTGATGAGCCT	AGCTGGACTTCCACCATCGCTA
STIM1	TGGATGAGGAGATTGTGTCGCC	GACTCCGAATCGGAATGGGTCA
TRPC1	TGGCAGTGAAGTTCCTCGTGGT	GGAAAATGGTGAAGGAGGCTGC
TRPC4	CTTGAACAGGCAAGGTCCACCA	TGTAATCCTGGAGTCCGCCATC
TRPC6	TGGCAGTGAAGTTCCTCGTGGT	GGAAAATGGTGAAGGAGGCTGC
TRPV4	TCACCGCCTACTATCAGCCACT	GAACAGGACTCCTGTGAAGAGC
PKD2	TCTACTCCGTCAGCAGTGAGGA	GCTCCACTGTACGACGCAATGA

Reference

Ballard, D. H. (1981). Generalizing the Hough transform to detect arbitrary shapes. *Pattern Recognition, 13*(2), 111-122.

Liu, Y., Mollaeian, K., & Ren, J. (2018). An image recognition-based approach to actin cytoskeleton quantification. *Electronics, 7*(12), 443.

Yang, M. T., Fu, J., Wang, Y.-K., Desai, R. A., & Chen, C. S. (2011). Assaying stem cell mechanobiology on microfabricated elastomeric substrates with geometrically modulated rigidity. *Nature protocols, 6*(2), 187-213.
